# *Artemisia argyi* extract alleviates inflammation in a DSS-induced colitis mouse model and enhances immunomodulatory effects in lymphoid tissues

**DOI:** 10.1186/s12906-022-03536-x

**Published:** 2022-03-11

**Authors:** Ji Min Shin, Yang-Ju Son, In Jin Ha, Saruul Erdenebileg, Da Seul Jung, Dae-geun Song, Young Sik Kim, Sang Min Kim, Chu Won Nho

**Affiliations:** 1 Smart Farm Research Center, Korea Institute of Science and Technology (KIST), Gangneung Institute of Natural Products, Gangneung, 25451 Gangwon-do Korea; 2 Department of Food and Nutrition, College of Biotechnology and Natural Resources, Chung-Ang University, Anseong, 17546 Korea; 3 Korean Medicine Clinical Trial Center (K-CTC), Kyung Hee University Korean Medicine Hospital, Seoul, 02454 Korea; 4 Division of Bio-Medical Science & Technology, KIST School, Korea University of Science and Technology, Seoul, 02792 Korea; 5 Natural Products Informatics Center, Korea Institute of Science and Technology (KIST), Gangneung Institute of Natural Products, Gangneung, 25451 Gangwon-do Korea; 6 College of Pharmacy, Natural Products Research Institute, Seoul National University, Seoul, 08826 Korea

**Keywords:** Inflammatory bowel diseases, *Artemisia argyi*, Natural products, Spleen, UPLC-MS, MS

## Abstract

**Background:**

The incidence of inflammatory bowel disease (IBD), an inflammatory disorder of the gastrointestinal system has increased. IBD, characterized by aberrant immune responses against antigens, is thought to be caused by the invasion of enterobacteria. The pathogenesis of IBD is complicated, hence novel effective therapeutic agents are warranted. Therefore, this study evaluates the potential of *Artemisia argyi*, a medicinal herb, in alleviating IBD.

**Methods:**

The effectiveness of the *A. argyi* ethanol extract was verified both in vitro and in vivo*.* Inflammation was induced in RAW 264.7 cells by 1 μg/mL of lipopolysaccharide (LPS) and by 3% dextran sodium sulfate (DSS) in a DSS-induced colitis mouse model. During the ten-day colitis induction, 200 mg/kg of *A. argyi* ethanol extract was orally administered to the treatment group. Levels of inflammation-related proteins and genes were analyzed in the colon, serum, and lymphoid tissues, i.e., Peyer’s patches (PPs) and spleen. The chemical constituent of the *A. argyi* ethanol extract was identified using an ultra-high performance liquid chromatography mass spectrometry (UPLC-MS/MS) analysis.

**Results:**

*A. argyi* ethanol extract treatment ameliorated IBD symptoms and reduced the expression of inflammation-related proteins and genes in the colon and serum samples. Furthermore, *A. argyi* treatment induced the activation of anti-oxidative associated proteins, such as nuclear factor-erythroid factor 2-related factor 2 (Nrf2) and heme oxygenase-1 (HO-1); and the treatment have also inhibited nuclear factor-κB (NF-κB), a central mediator of inflammatory responses. *A. argyi* enhanced the immunomodulatory effects in the PPs and spleen, which may stem from interleukin-10 (IL-10) upregulation. Chemical analysis identified a total of 28 chemical compounds, several of which have been reported to exert anti-inflammatory effects.

**Conclusions:**

The effectiveness of the *A. argyi* ethanol extract in alleviating IBD was demonstrated; application of the extract successfully mitigated IBD symptoms, and enhanced immunomodulatory responses in lymphoid tissues. These findings suggest *A. argyi* as a promising herbal medicine for IBD treatment.

**Supplementary Information:**

The online version contains supplementary material available at 10.1186/s12906-022-03536-x.

## Background

The incident of inflammatory bowel disease (IBD), which includes ulcerative colitis (UC) and Crohn’s disease (CD), has gradual increased worldwide [1]. IBD incidence has particularly increased in Asian countries since the 1980s [2]. The pathogenesis of IBD is complex, but one known cause is an aberrant immune response to luminal antigens [3], linked to the complex interaction of various factors including the immune response, gut microbiota, environment, genetic factors, and psychological factors such as anxiety, depression, and stress [4]. While the mechanism underlying IBD pathogenesis remains nebulous, several studies have reported that IBD is responsible for the immunological imbalances of the intestinal mucosa [5, 6]. Shih and Targan reported that a mucosal susceptibility or the abnormal action of luminal antigen may induce an innate immune response, mediated by intensified activity of Toll-like receptor (TLR), leading to the stimulation of dendritic cells (DC) and subsequent abnormal differentiation of naive T-cells into effector T-cells [6].

The intestinal immune response is moderated via Peyer's patches (PPs), which are primarily distributed in the ileum region of the small intestine [7]. The characteristics of uncontrolled innate and adaptive immune responses are frequently observed in PPs of IBD patients [8]. In addition, although IBD is primarily associated with the gastrointestinal region, evidence has suggested the involvement of the spleen [9]. The spleen consists of an assemblage of lymphoid tissue, including red pulp, which filters pathogens and aged erythrocytes, and white pulp, a lymphoid region with adaptive immune response function [10]. However, the exact relationship between these immune tissues has not been verified, despite their reported involvement in IBD.

Treatment strategies for IBD have been developed [11]. The universal therapeutic agents of IBD, which aggressively target the characteristic inflammation, include 5-aminosalicylates (5-ASA), mesalazine, immunosuppressants, and biologics such as anti-TNF [11, 12]. However, these treatments are accompanied by several adverse effects, including toxicity, skin lesions, and immune dysfunction [13, 14]. More recently, microbiome modulators, including antibiotics and probiotics, have been introduced [15], albeit with mixed success, as gut microbiota population dynamics are not well understood. Consequently, therapeutic agents with relatively few side effects and increased efficacy remain warranted, of which medicinal herbs have been proposed as candidates. In particular, multilateral effects of medicinal herbs have been suggested to be commensurate with the complex pathogenesis of IBD, possibly allowing alteration of the gut microbiome as well as the enhancement of the innate immune system, which may go beyond the traditional treatments, which only aim to reduce inflammation [16].

*Artemisia argyi*, also known as wormwood and Chinese mugwort, is a traditional herb used in East Asia to treat inflammatory diseases such as hepatitis, gastric ulcer, and dysmenorrhea [17, 18]. In particular, the epigeal parts of *A. argyi* are a common food ingredient in Korea [19]. Additionally, the extract of *A. argyi* and its chemical components have antitumor effects on several cell lines [20]. However, the inflammatory and immune-related activities of *A. argyi* in a dextran sodium sulfate (DSS)-induced colitis mouse model have yet to be determined. Thus, in this study, we investigate the effect *A. argyi* in mitigating the inflammatory activity in colon tissue using the DSS-induced colitis mouse model. We also investigate the inflammatory responses in both spleen and PPs to determine the association between these tissues and IBD.

## Methods

### Plant materials

*A. argyi* plant samples were obtained from Ganghwa Agricultural Technology Service Center (Incheon, Korea). The identification of *A. argyi* was attended by Ganghwa Agricultural Technology Service Center in advance and the center supervises purity of *A. argyi* that cultivated in Ganghwa district. We obtained *A. argyi* samples which cultivated in Ganghwa district in 2018, and its cultivation was conducted following the regulation of Korea. In addition, the farm also has authorization from the Korean government to distribute cultivated *A. argyi* plants in public. Thus, the collection of *A. argyi* was conducted under valid permission. The obtained *A. argyi* was identified by Dr. In Jin Ha (Kyung Hee University Korean Medicine Hospital), and the voucher specimen is deposited in Gangneung Institute of Natural Products, Korea Institute of Science and Technology (voucher NO. KIST-2018–003). An extract of *A. argyi* in ethanol was prepared using 100 g of dried *A. argyi* (epigeal part was prepared) powder was mixed with 1 L of ethyl alcohol (1:10, w/v) and extracted with a shaker (SI600R; Lab Companion, Korea) for 12 h at 20 ℃. The supernatant was collected after filtration, and the extraction procedure was repeated two additional times more using the same powder. The collected liquids were then concentrated using a Rotavapor R-100 (Büchi, Flawil, Switzerland).

### Cell cultures

RAW 264.7 cell line (mouse leukemia macrophage cell) was obtained from American Type Culture Collection (Manassas, VA, USA). The RAW 264.7 cells were cultured in minimum essential media (MEM) supplemented with 1% antibiotics and 10% fetal bovine serum (Sigma-Aldrich, St. Louis, MO, USA). The cells were incubated in a 5% CO_2_ incubator at 37 ℃.

### Nitric oxide (NO) assay

RAW 264.7 cells were seeded onto a 96-well plate at a density of 1 × 10^4^ cells/well and incubated for 24 h. The culture media was then removed, and fresh media with a range of concentrations of A. argyi extract was added to the wells (50 µL; ranged from 0 to 160 μg/mL); 30 min later, 50 µL of media containing lipopolysaccharide (LPS; 2 μg/mL) was added to the treatment group and incubated for 24 h. Next, the cultured supernatant was transferred to fresh plate; its NO concentration was then detected using the Griess assay (Sigma-Aldrich) and Synergy multi-plated reader (BioTek, Winooski, VT, USA).

### Preparation of the nuclear and cytosolic fraction

RAW 264.7 cells were plated on 6-well plate at a density of 1.6 × 10^5^ cells/well and incubated for 24 h. The culture media was then removed, and fresh media with a range of concentrations of *A. argyi* extract was put into the wells. After incubation for 24 h, the nuclear and cytosolic fractions were separately collected using Cayman’s Nuclear Extraction Kit (Cayman, Ann Arbor, MI, USA) following manufacturer’s instructions.

### Animal study

Seven-week-old C57/BL6 male mice were obtained from Orient bioscience (Seongnam, Korea) and were acclimatized for five days immediately preceding the experiment, and were housed in a specific pathogen-free room with filter-top cages under 12 h light:dark cycle and allowed to free access to AIN-76A diet and water. This animal experiment was approved by the International Animal Care and Use Committee of Korea Institute Science and Technology (Approval NO.: KIST-2019–060), and in accordance with the Animal Research: Reporting of In Vivo Experiments (ARRIVE) guidelines of National Centre for the Replacement Refinement & Reduction of Animals in Research (NC3Rs; London, United Kingdom). In order to establish a DSS-induced colitis mouse model, acute colitis was induced by providing 3% DSS in drinking water to all mice groups except the CON group for 10 days. Before providing pure water or 3% DSS solution to each mice groups, the mice were randomly divided into following 4 groups (*N* = 8 for each group): the CON and DSS group, treated with a 0.5% carboxymethyl cellulose vehicle solution (CMC), the ASA group, treated with 100 mg/kg of 5-ASA in the vehicle solution, and the AA group, treated with 200 mg/kg ethanol extract of *A. argyi* in the vehicle solution. Administration of all treatments was conducted by oral gavage feeding once per day during the entire colitis-inducing period. Body weight, condition of feces, and rectal bleeding were monitored daily for all mice. The Disease Activity Index (DAI) for the collected mouse feces was graded for stool consistency (0, normal; 2–3, loose stools; 4, diarrhea) and visible blood in feces (0, none; 1, visible blood; 2, slight bleeding; 3, gross bleeding; 4, bleeding diarrhea) [21].

### Primary cell culture of PPs

PPs were isolated from mouse small intestines and immediately placed into primary cell culture medium consisting of RPMI 1640 medium supplemented with 1 mM sodium pyruvate, non-essential amino acids, and antibiotics (Sigma-Aldrich). To isolate the PPs as single cells, PPs were filtered through a cell strainer and the primary cells from PPs were seeded in a 48-well culture plate at a density of 2 × 10^6^ cells/well and incubated for 3 days, after which the supernatant of the culture medium was prepared using centrifugation at 16,900 × *g* for 10 min at 4 ℃. The protocols were slightly modified from a previous method [22].

### Hematoxylin and eosin (H&E) staining

The isolated colon tissue sections and spleen tissues were fixed and embedded on paraffin blocks. Deparaffinized slides were subsequently stained with H&E. The protocols were slightly modified from previously described protocols [23]. The histological images were detected using an Axio Zoom Carl Zeiss microscope (Oberkochen, Germany).

### Enzyme-linked immunosorbent assay (ELISA) and colorimetric assay

The levels of interleukin-6 (IL-6), IL-1β, and tumor necrosis factor-α (TNF-α) in mouse serum were measured using commercial kits (RayBiotech, Peachtree Corners, GA, USA) following the manufacture’s recommendation. Myeloperoxidase (MPO) and prostaglandin E_2_ (PGE_2_) ELISA kits were purchased from BioVision (Milpitas, CA, USA). To determine the levels of aspartate aminotransferase (AST) and alanine aminotransferase (ALT) in mouse serum, we performed colorimetric analysis using commercial kits (ElabScience, Houston, TX, USA).

### Western blot assay

RAW 264.7 cells were cultured as described above, and the cell lysate was prepared using an ice-cold radioimmunoprecipitation assay buffer (Thermo Fisher Scientific, Waltham, MA, USA) containing a protease inhibitor cocktail and phenylmethane sulfonyl fluoride (Sigma-Aldrich). Mice colon samples were homogenized using the Bio-Masher II (Optima, Tokyo, Japan). The protein concentration was measured using the Bradford Protein Assay Dye Reagent (Bio-Rad, Hercules, CA, USA), and the total cell lysate, nuclear and cytosolic lysates were used for Western blot analysis as previously described [24]. The antibodies used were: β-actin, Lamin B, cyclooxygenase-2 (Cox2), heme oxygenase-1 (HO-1) (Santa Cruz Biotechnology, Dallas, TX, USA); nuclear factor-erythroid factor 2-related factor 2 (Nrf2) (Abcam, Cambridge, MA, USA); phospho-nuclear factor-κB (p-NF-κB), NF-κB, p-IκBα, TNF-α, inducible nitric oxide synthase (iNOS) (Cell Signaling Technology, Danvers, MA, USA). Anti-rabbit and anti-mouse secondary antibodies were obtained from Santa Cruz (USA). Proteins were detected using SuperSignal™ West Femto Maximum Sensitivity Substrate (Thermo Fisher Scientific) and detected using the LAS 4000 with Multi Gauge 3.1 software (Fujifilm, Tokyo, Japan). The protein band was measured using ImageJ software (NIH, Bethesda, MD, USA).

### RNA extraction and quantitative real-time polymerase chain reaction (qRT-PCR)

To isolate RNA from RAW 264.7 cells, cultured cells were washed using cold phosphate-buffered saline (PBS) and harvested with a Hybrid-R RNA Isolation Kit (GeneAll, Seoul, Korea). Frozen colon, PPs, and splenic tissues were extracted using same kit. cDNA was synthesized using a PrimeScript cDNA Synthesis Kit (Takara, Shiga, Japan) following the manufacturer’s protocol. qRT-PCR was performed using Power SYBR Green Master Mix (Thermo Fisher Scientific) and detected using a Light Cycler 480 (Roche, Basel, Switzerland). The sequences of primers are presented in Additional File 1.

### Immunohistochemistry

Spleen tissue sections on slides were prepared from embedded paraffin blocks via deparaffinizing. After blocking using 5% bovine serum albumin (BSA) in 0.1% Tween 20 in PBS solution for 30 min, slides were incubated in primary antibody solution overnight at 4 °C, then washed three times. The tissue sections were then stained with Alexa Fluor 488-conjugated anti-mouse and Alexa 594-conjugated anti-rabbit secondary antibodies (Thermo Fisher Scientific). After washing five times for 5 min each, slides were treated with VECTASHIELD Antifade Mounting Medium with DAPI (Vector Laboratories, Burlingame, CA, USA) to preserve fluorescence. Images were acquired using a Nikon TE2000-U fluorescence microscope (Nikon, Kanagawa, Japan).

### Chemical profiling of *A. argyi* using an ultra-high performance liquid chromatography-quadrupole time-of-flight tandem mass spectrometry (UPLC-QTOF-MS/MS)

Chemical profiling of the *A. argyi* ethanol extract was obtained via UPLC-MS/MS analysis. Ten milligrams of *A. argyi* extract was mixed with 1 mL of 50% ethanol solution, and its supernatant was filtered using a 0.2 μm syringe filter (Thermo Fisher Scientific). The ACQUITY UPLC HSS T3 column (2.1 mm × 100 mm, 1.8 μm; Waters, Milford, MA, USA) was equipped to Thermo Scientific Vanquish UPLC system (Thermo Fisher Scientific) to separate the chemical compounds within *A. argyi* extract. The Triple TOF 5600^+^ mass spectrophotometer (Triple TOF MS; QTOF, SCIEX, Foster City, CA, USA) and electrospray ionization (ESI) method was used for MS/MS analysis. The mobile solvents were water containing 0.1% formic acid (A) and acetonitrile containing 0.1% formic acid (B); their mobile gradient was as follows: 0–1 min, 95:5; 4 min, 85:15; 11 min, 65:35; 17 min, 50:50; 19 min, 0:100; 19––24 min, 0:100, 28 min, 95:5 (A:B). The flow rate of the mobile solvent was 0.4 mL/min, and the injection volume was 2 μL. The column temperature was maintained for 40 ℃ and the inner temperature of the auto-sampler was 4 ℃. The Analyst TF 1.7, PeakView 2.2 and Master View (SCIEX) systems were used for acquisition and processing for mass spectrometric data. The MS/MS signals of each chemical compound in *A. argyi* extract were processed using PeakView and MasterView software to identify known compounds or to search for characterization of chemical compounds putatively.

### Statistical analysis

Data are represented as the mean and standard error of the mean (SEM). Differences in mean values were assessed via one-way analysis of variance (ANOVA) with Duncan’s multiple comparison test using Prism version 7 software (GraphPad, San Diego, CA, USA) and SPSS V.25 (IBM SPSS, Chicago, IL, USA).

## Results

### Effects of *A. argyi* extract on inflammatory activity in LPS-induced RAW 264.7 cells

**Fig. 1:**
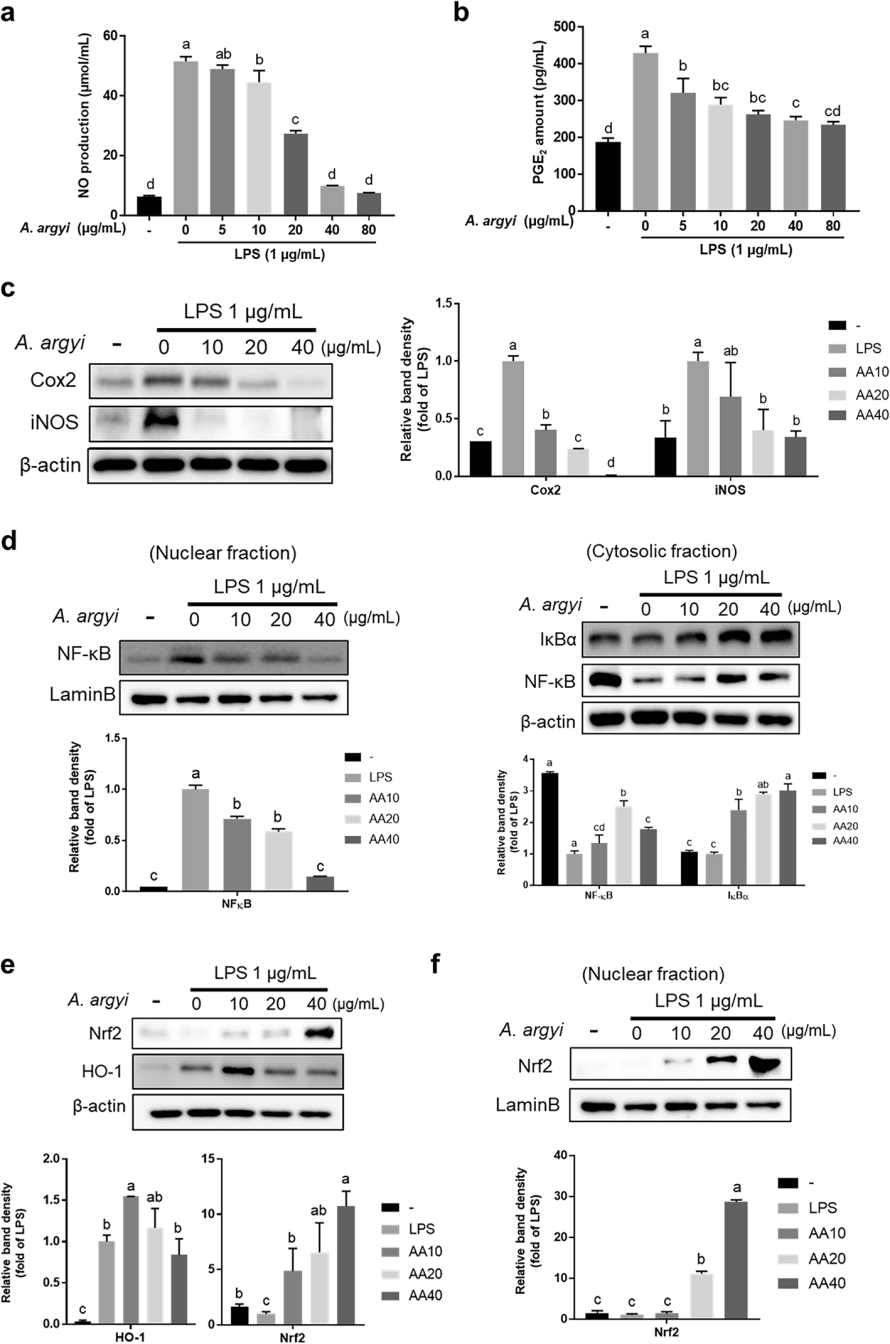
Anti-inflammatory and anti-oxidative effect of *A. argyi* on LPS-induced RAW 264.7 cells. **a**, **b** Amounts of nitric oxide (NO) and prostaglandin E_2_ (PGE_2_) were measured using the supernatant of RAW 264.7 cells. Cells were treated with the *A. argyi* extract (0–80 μg/mL) for 24 h. **c** Protein expression levels of Cox2 and iNOS were determined using western blotting in LPS-induced RAW 264.7 cells. The cells were treated with 0, 10, 20, and 40 μg/mL of the *A. argyi* extract for 24 h. **d** The protein expression level of NF-κB accumulation in the nucleus. The cells were treated with the *A. argyi* extract (0, 10, 20, and 40 μg/mL) for 24 h. **e**–**f** The protein expression levels of Nrf2 and its target gene HO-1 were determined using western blotting in LPS-induced RAW 264.7 cells. The cells were treated with 0, 10, 20, and 40 μg/mL of the *A. argyi* extract for 24 h. Data are presented as mean ± standard error of the mean (SEM) from three independent experiments. Different superscripts indicate significant differences at *P* < 0.05

To examine the anti-inflammatory effects of *A. argyi* extract, we performed an NO assay using LPS-induced RAW 264.7 murine leukemia macrophage cells. As shown in Fig. 1a-b, the NO production and the level of PGE_2_ decreased with *A. argyi* extract treatment in a dose-dependent manner. *A. argyi* extract also decreased the protein expression of iNOS and Cox2 (Fig. 1c). Moreover, to determine whether *A. argyi* extract regulates NF-κB nuclear translocation, we assessed the translocation and expression of IκBα and NF-κB. In the nuclear fraction, NF‐κB induced by LPS was decreased with *A. argyi* extract treatment in a dose-dependent manner, while the cytosolic IκBα and NF‐κB protein contents were elevated by *A. argyi* extract due to decreased translocation (Fig. 1d). In addition, we investigated the involvement of Nrf2 pathway proteins due to the participation of inflammation-related factors, including cytokines, Cox2, and iNOS, in the NF‐κB signaling pathway [25]. *A. argyi* extract treatment increased the protein expression of Nrf2 and HO-1, and the nuclear translocation of Nrf2 also increased with *A. argyi* extract treatment in a dose-dependent manner (Fig. 1e-f). Altogether, treatment with the extract of *A. argyi* reduced the inflammatory responses through the regulation of NF-κB and Nrf2 pathway *in vitro*.

### Effects of *A. argyi* extract on DSS-induced colitis symptoms in mice

**Fig. 2:**
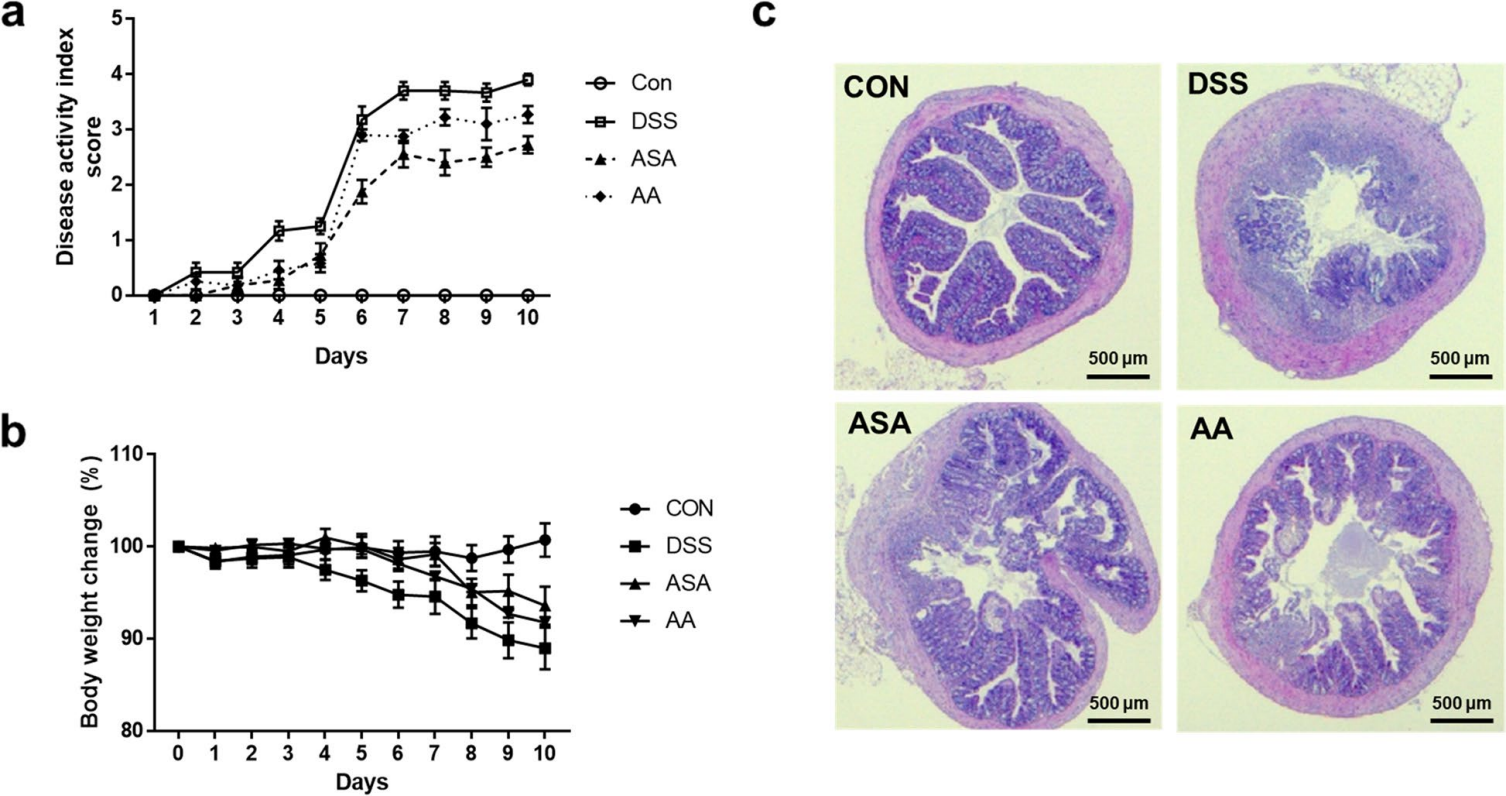
The ethanol extract of *A. argyi* ameliorated colitis symptoms in a dextran sodium sulfate (DSS)-induced colitis model. **a** The disease activity index (DAI) score was measured using mouse feces. **b** Changes in body weights of four mouse groups. **c** The histological images of colon tissue. Colon tissue sections were stained with H&E (scale bar = 500 μm). CON, the mice group was provided pure water; DSS, the mice group was provided 3% DSS solution; ASA, the mice group was treated with 3% DSS solution and 100 mg kg-1 day-1 of 5-amino salicylic acid (5-ASA); AA, the mice group was treated with 3% DSS solution and 200 mg kg-1 day-1 of A. argyi extract

Oral administration of DSS can cause severe colitis in mouse models [26]. In this study, to establish an acute colitis model, we provided 3% DSS solution to mice up to ten days. The DSS group showed most severe DAI score; however, the severity of IBD was abated by administration of *A. argyi* and 5-ASA (Fig. 2a). The AA and ASA group also showed reduced loss of body weight compared with that in the DSS group (Fig. 2b), suggesting mitigated IBD severity. In addition, we found that the colon dysplasia and disruption of the colon barrier was also ameliorated in the AA and ASA group (Fig. 2c). Our results therefore showed that *A. argyi* extract treatment relieved symptoms and pathogenesis of acute colitis including DAI, body weight loss, and histological changes in a DSS-induced colitis animal model.

### Anti-inflammation and anti-oxidative capacities of *A. argyi* in DSS-induced colitis mice

**Fig. 3:**
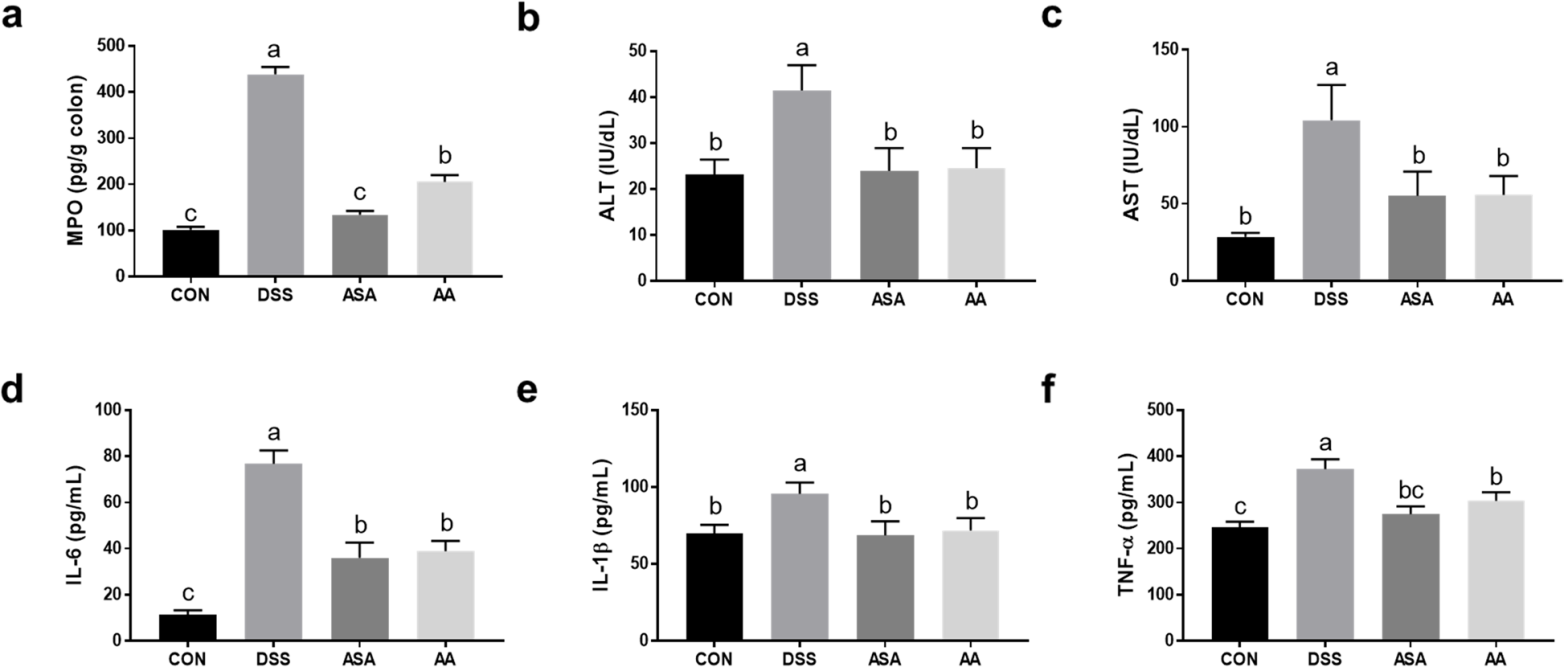
The A. argyi extract reduced the levels of pro-inflammatory cytokines in mice with DSS-induced colitis. **a** Myeloperoxidase (MPO) activity. **b**, **c** The level of alanine aminotransferase (ALT) and aspartate aminotransferase (AST) in mouse serum. **d**-**f** The contents of pro-inflammatory cytokines, including IL-6, IL-1β, and TNF-α, in serum. Data are presented as mean ± SEM from three independent experiments. Different superscripts indicate significant differences at *P* < 0.05. CON, the mice group was treated with pure water; DSS, the mice group was treated with 3% DSS solution; ASA, the mice group was treated with 3% DSS solution and 100 mg kg-1 day-1 of 5-amino salicylic acid (5-ASA); AA, the mice group was treated with 3% DSS solution and 200 mg kg-1 day-1 of A. argyi extract

MPO is an enzyme that is released from the phagosomes of neutrophils, and it is generally used as a biomarker to determine the level of colon inflammation in IBD [27]. We found that the 5-ASA and *A. argyi* dramatically decreased MPO activity compared to the DSS group (*P* < 0.05; Fig. 3a). To confirm the cytotoxic effect of colitis in mouse liver, we measured the ALT and AST in mouse serum. The treatment of *A. argyi* and 5-ASA significantly reduced both ALT and AST levels compared with those in the DSS group (*P* < 0.05; Fig. 3b-c). This indicated that outbreaks of IBD place a burden on the liver and that *A. argyi* and 5-ASA could therefore have a protective effect on the liver. Moreover, the AA and ASA groups showed significantly decreased IL-6, IL-1β, and TNF-α contents in serum compared to the DSS group (*P* < 0.05; Fig. 3d-f).

**Fig. 4:**
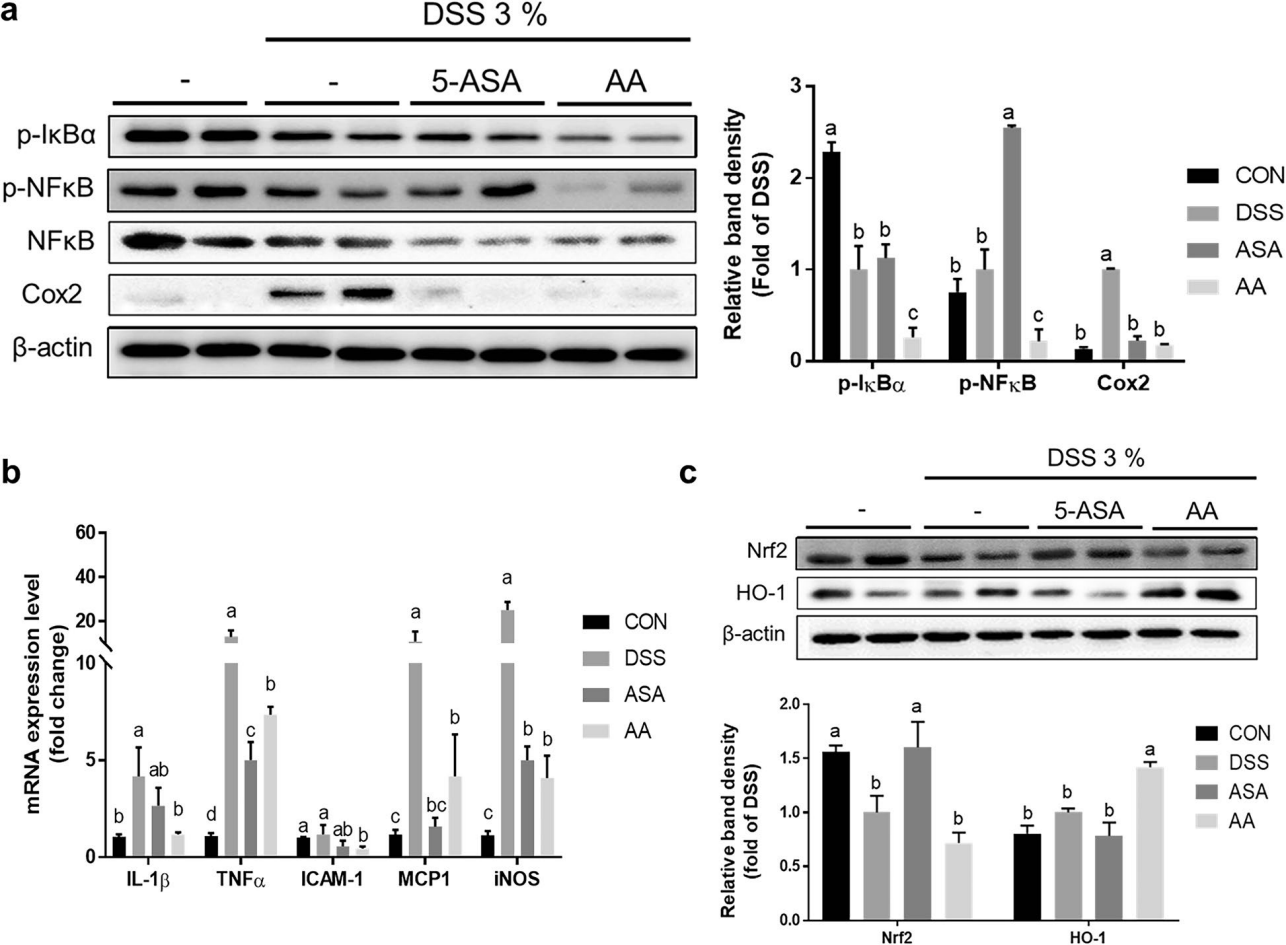
Inflammatory marker levels decreased following *A. argyi* extract treatment in mice with DSS-induced colitis. **a** The protein expression levels of inflammation-related markers, including phospho-IκB and phospho-NF-κB, and Cox2, were determined using western blotting in mouse colon tissue. Band densities of phospho-IκB and Cox2 were normalized to that of β-actin and the density of phospho-NF-κB was normalized to that of NF-κB. **b** mRNA expression of inflammation-related genes, including *IL-1β, TNF-α, ICAM-1, MCP1*, and *iNOS*. The mRNA level was normalized using *GAPDH*. **c** The protein expression levels of Nrf2 and its target protein HO-1 were detected using western blotting in mouse colon tissue. Band densities of Nrf2 and HO-1 were normalized to that of β-actin. Data are presented as mean ± SEM from three independent experiments. Different superscripts indicate significant differences at *P* < 0.05. CON, the mice group was treated with pure water; DSS, the mice group was treated with 3% DSS solution; ASA, the mice group was treated with 3% DSS solution and 100 mg kg-1 day-1 of 5-amino salicylic acid (5-ASA); AA, the mice group was treated with 3% DSS solution and 200 mg kg-1 day-1 of A. argyi extract

To determine whether *A. argyi* extract regulates inflammation-related factors in vivo, we examined the expression levels of inflammatory response related proteins. We found that the ethanol extract of *A. argyi* significantly reduced the levels of inflammatory protein markers, including p-IκBα, p-NF‐κB, and Cox2 (Fig. 4a). In addition, mRNA expression levels of inflammatory genes, such as *IL-1β*, *TNF-α*, and intercellular adhesion molecule 1 (*ICAM-1*), monocyte chemoattractant protein-1 (*MCP1*) and *iNOS*, were significantly decreased in the AA group compared to those in the DSS group (*P* < 0.05; Fig. 4b). Although the Nrf2 protein expression level in the AA group was not significantly increased compared to that in the DSS group, we observed a significant increase in the HO-1 protein level in the AA group, suggesting that it may be a receptor for the target gene Nrf2 (*P* < 0.05; Fig. 4c).

### Changes in the immune response in mouse PPs and spleen following *A. argyi* administration

**Fig. 5:**
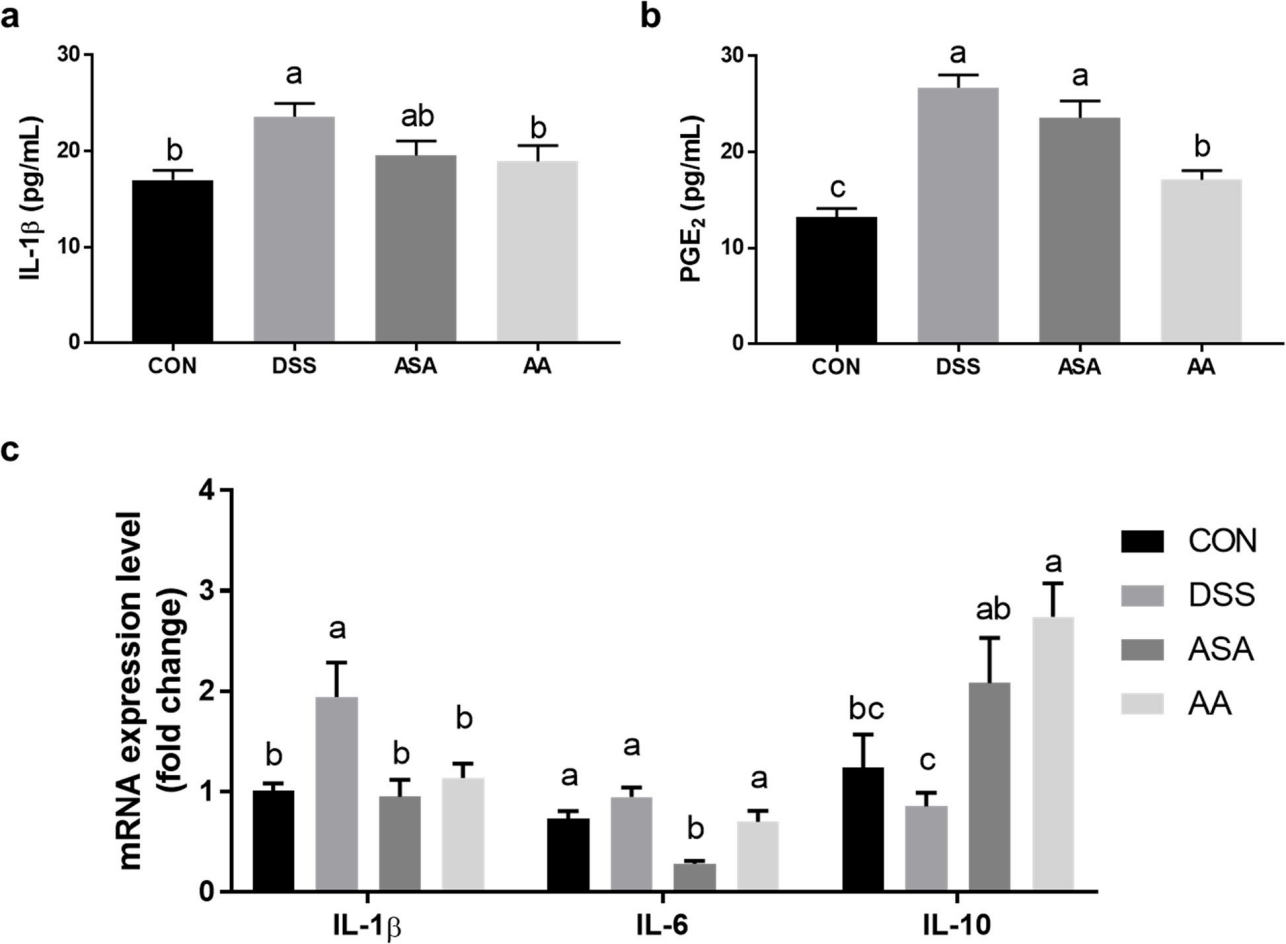
***A. argyi*** ethanol extract ameliorated inflammatory responses in Peyer’s patches from mice with DSS-induced colitis. **a**, **b** Levels of pro-inflammatory cytokines (IL-1β and PGE_2_) were determined using the supernatant of primary cultured cells derived from mouse Peyer’s patches. **c** mRNA expression levels of inflammation-related genes, including *IL-1β, IL-6*, and *IL-10,* in Peyer’s patches. The mRNA levels were normalized to *GAPDH* levels. Data are presented as mean ± SEM from three independent experiments. Different superscripts indicate significant differences at *P* < 0.05. CON, the mice group was treated with pure water; DSS, the mice group was treated with 3% DSS solution; ASA, the mice group was treated with 3% DSS solution and 100 mg kg-1 day-1 of 5-amino salicylic acid (5-ASA); AA, the mice group was treated with 3% DSS solution and 200 mg kg-1 day-1 of A. argyi extract

IBD pathogenesis is not specific to the intestine, as it involves immune responses in related organs [28]. Therefore, we verified the expression level of pro-inflammatory cytokines on PPs and spleen tissue to examine the immunoregulatory effects of *A. argyi* in lymphoid tissues. Using the supernatant from a primary cell culture media of PPs, we measured IL-1β and PGE_2_ levels (Fig. 5a-b). Both protein levels were significantly decreased in the AA group, along with the mRNA expression of IL-1β and IL-6 in the PPs (Fig. 5c), and the level of IL-10 was significantly elevated in the AA group (*P* < 0.05).

**Fig. 6:**
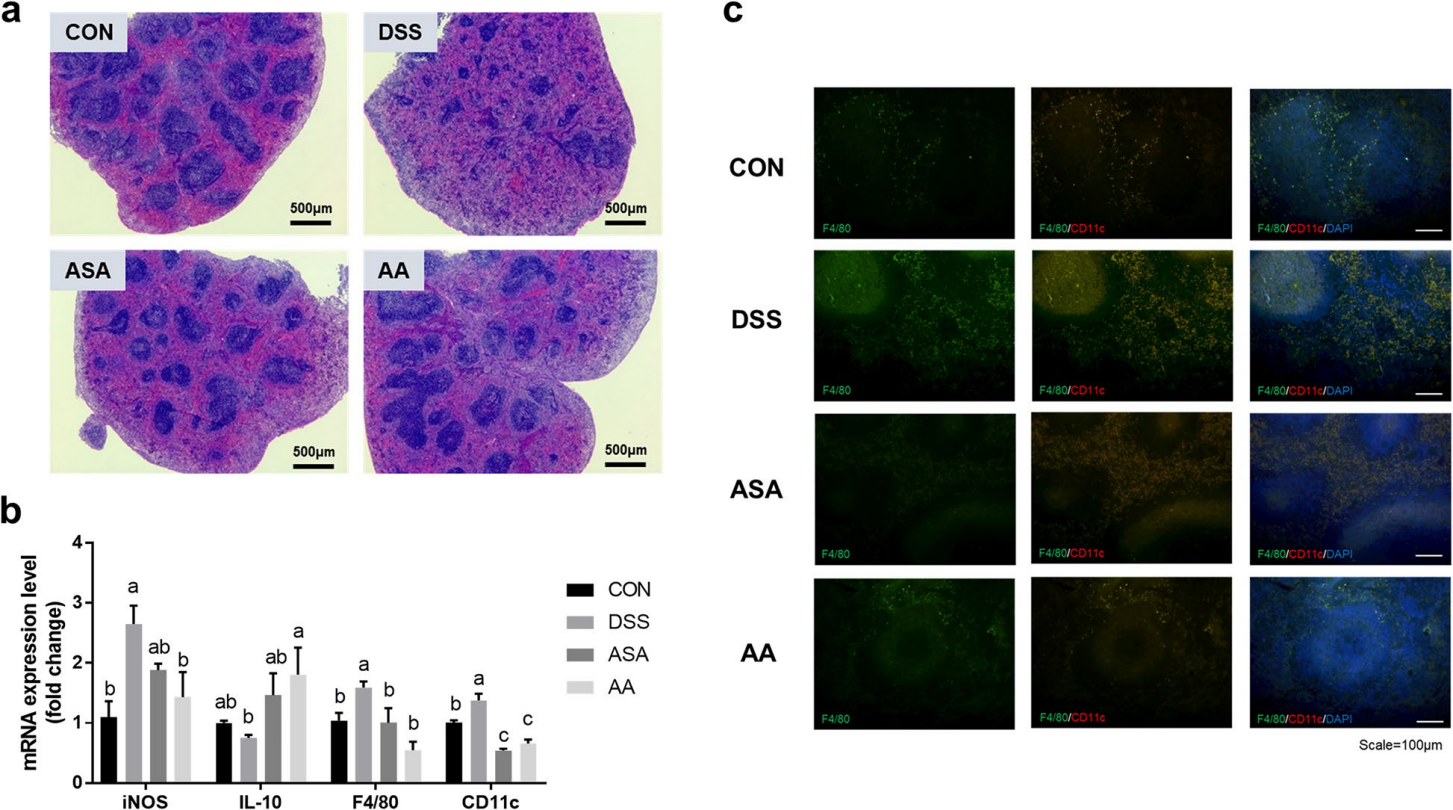
*A. argyi* treatment enhanced immune responses in the splenic tissue of mice with DSS-induced colitis. **a** The histological image of spleen tissue. Mouse spleen tissue sections were stained with H&E (scale bar = 500 μm). **b** mRNA expression of inflammation-related markers and M1 macrophage markers (*iNOS, IL-10, F4/80*, and *CD11c*). The mRNA level was normalized to that of *GAPDH*. **c** Immunohistochemistry against F4/80 and CD11c was performed in mouse splenic tissue (scale bar = 100 μm). Data are presented as mean ± SEM from three independent experiments. Different superscripts indicate significant differences at *P* < 0.05. CON, the mice group was treated with pure water; DSS, the mice group was treated with 3% DSS solution; ASA, the mice group was treated with 3% DSS solution and 100 mg kg-1 day-1 of 5-amino salicylic acid (5-ASA); AA, the mice group was treated with 3% DSS solution and 200 mg kg-1 day-1 of A. argyi extract

To determine whether *A. argyi* extract ameliorates splenic disorder, we checked the histology of mouse spleen using H&E staining images (Fig. 6a). The spleen consists of areas of white pulp and red pulp that are responsible for immune response and role of filtration with blood, respectively. Both structures were clearly present in the CON group, but the DSS group showed both morphology and boundaries disruption. Compared to the DSS group, the AA and ASA groups maintained the original structural properties of the spleen; therefore, we concluded that both *A. argyi* extract and 5-ASA ameliorated excessive immune responses and associated damage in spleen tissue. In the result of inflammation-related gene expression in spleen tissue, *iNOS*, *F4/80*, and *CD11c* levels decreased significantly in the AA group compared to those in the DSS group (*P* < 0.05; Fig. 6b). The inhibition of F4/80 and CD11c protein expression was also verified using immunohistochemistry (Fig. 6c), and the AA group showed obviously decreased CD11c^+^F4/80^+^ cells via tissue section. Conversely, the mRNA expression of *IL-10* was doubled in the AA group compared to that in the DSS group.

### Chemical profiling analysis of *A. argyi* extract

**Fig. 7:**
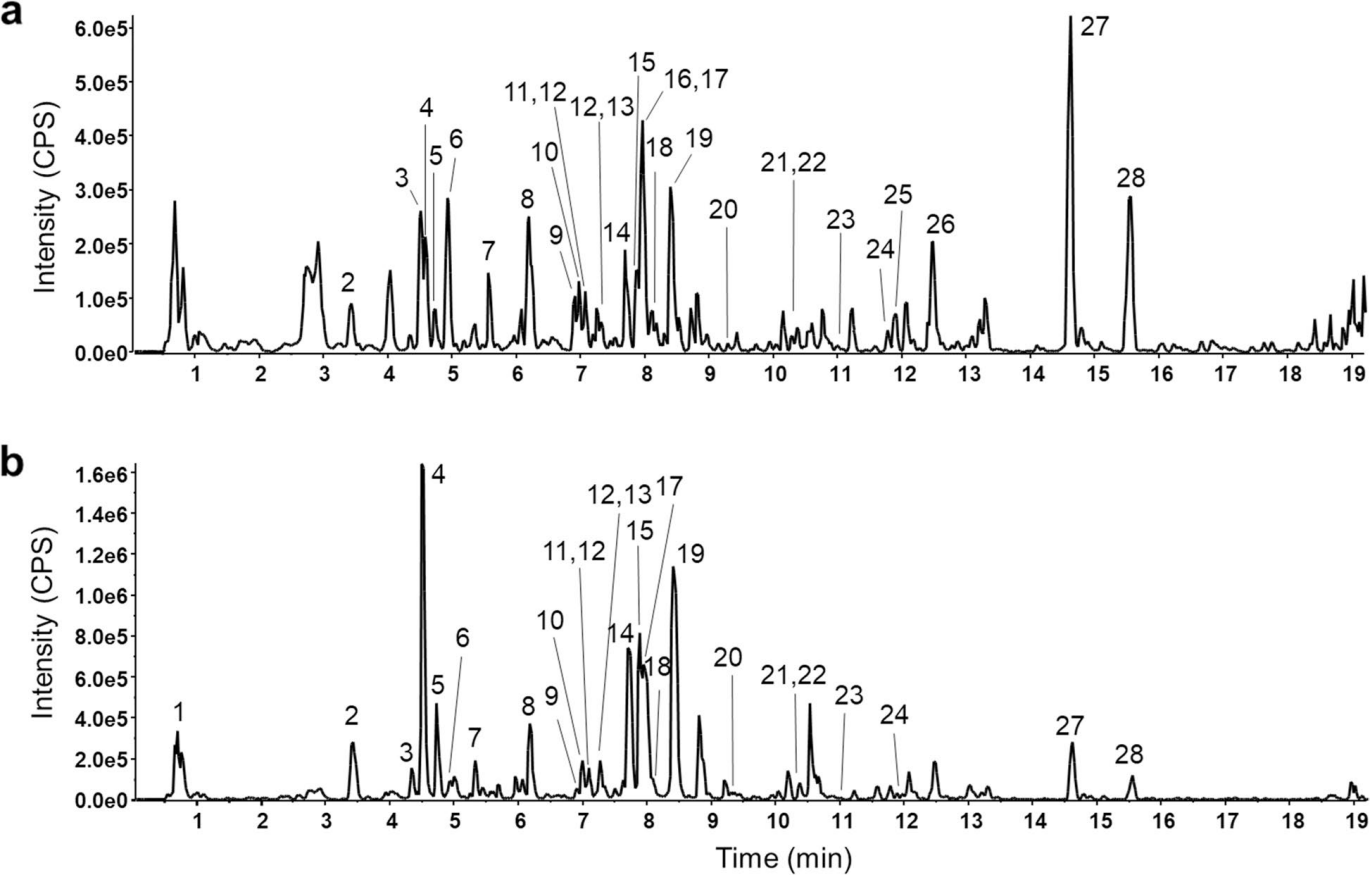
Representative base peak chromatograms of the *A. argyi* ethanol extract. **a** Positive ion mode and **b** negative ion mode were employed for analyzing the ethanol extract of *A. argyi*. Ultra-high-performance liquid chromatography quadrupole time-of-flight tandem mass spectrometry (UPLC-QTOF-MS/MS) was used for examination

The chemical profiling of *A. argyi* ethanol extract was determined using UPLC-QTOF-MS/MS analysis; its total ion current chromatogram is presented in Fig. 7. In addition, the results of the mass spectrometry analysis and the identified compound names are shown in Table 1, and the mass spectra of product ions are presented in Additional File 2. As a result, a total of 28 chemical compounds were identified in *A. argyi* ethanol extract, including organic acids, polyphenols, phenolic acids, and flavonoids such as flavones, flavonols, and an isoflavone glucoside (genistein glucoside). Moreover, we identified two coumarin compounds (scopoletin and escultein) and a lactone (arteannuin B), and six isomeric compounds of dicaffeoylquinic acid (DCQA). We also identified several phenolic compounds in *A. argyi* ethanol extract, with similar chemical profile previously reported [19, 29]. In addition, the unique and famous bioactive flavones in *Artemisia* species (jaceosidin and eupatilin) were richly contained in *A. argyi* ethanol extract. We tested whether the major chemical compounds (3,5-DCQA, 4,5-DCQA, chlorogenic acid, eupatilin, and jaceosidin) found in *A. argyi* extract may have anti-inflammation effects (Additional File 3); all five chemical compounds showed anti-inflammatory effects in vitro, with eupatilin and jaceosidin presenting distinctly more potent effects than the DCQA family compounds. However, their respective effectiveness was notably lower than that of the *A. argyi* ethanol extract itself.

**Table 1:** Chemical profiling analysis results of *A. argyi* ethanol extract

No	Name	Formula	Mass (Da)	Expected	Adduct	Found at	Error	MS/MS product ions	Identified with
				**RT (min)**		**mass (Da)**	**(ppm)**		
1	Quinic acid	C7H12O6	192.0634	0.69	[M-H] ^−^	191.0564	1.4	85.0319, 127.0407, 93.0362, 173.0461, 171.0303	^#^
2	Neochlorogenic acid	C16H18O9	354.0951	3.42	[M + H] ^+^	355.1029	0.9	163.0394, 145.0285, 135.0443, 117.0339	^†^
					[M-H] ^−^	353.0873	-1.4	191.0558, 179.0348, 135.0453, 173.0448, 161.0240,155.0346, 134.0369	^†^
3	Chlorogenic acid	C16H18O9	354.0951	4.51	[M + H] ^+^	355.1030	0.7	163.0394, 145.0293, 135.0450	^†^
					[M-H] ^−^	353.0881	0.8	191.0562, 161.0247, 179.0355, 173.0459, 85.0309	^†^
4	Cryptochlorogenic acid	C16H18O9	354.0951	4.73	[M + H] ^+^	355.1028	0.5	163.0395, 145.0290, 135.0447, 117.0344	^†^
					[M-H] ^−^	353.0872	0.5	173.0455. 179.0349, 131.0560, 135.0454, 137.0240, 155.0348, 93.0357	^†^
5	Esculetin	C9H6O4	178.0266	4.88	[M + H] ^+^	179.0339	-1.1	123.0438, 133.0264, 77.0384, 151.0349, 135.0430	^†^
					[M-H] ^−^	177.0194	0.2	133.0291, 105.0352, 149.0242, 89.0410	^†^
6	Caffeic acid	C9H8O4	180.0423	4.99	[M + H] ^+^	181.0491	-2.3	169.0388, 135.0440, 145.0287, 117.0342, 89.0402, 107.0494	^†^
					[M-H] ^−^	179.0351	0.7	135.0453, 134.0376, 136.0483, 107.0506, 117.0352, 89.0416, 79.0569	^†^
7	1,5-dicaffeoylquinicacid	C25H24O12	516.1268	5.70	[M + H] ^+^	517.1341	0.2	163.0389, 499.1224, 337.0915,145.0283,135.0442	^†^
					[M-H] ^−^	515.1195	0.0	353.0879, 179.0343, 191.0558, 335.0763, 135.0447	^†^
8	Apigenin-C-hexoside-C-pentoside (tentatively Schaftoside or isoschaftoside)	C26H28O14	564.1479	6.19	[M + H] ^+^	565.1550	-0.4	547.1464, 427.1039, 511.1249, 529.1358, 409.0933, 379.0828	^#^
					[M-H] ^−^	563.1411	0.8	353.0682, 383.0787, 443.1005, 473.1114, 503.1221, 545.1333	^#^
9	scopoletin	C10H8O4	192.0423	6.90	[M + H] ^+^	193.0495	-0.8	178.0253, 133.0380, 137.0591, 150.0311, 122.0359	^†^
					[M-H] ^−^	191.0352	0.9	176.0114, 148.0164, 104.0274, 120.0215	^†^
10	Rutin	C27H30O16	610.1534	6.98	[M + H] ^+^	611.1604	-0.4	303.0510, 465.1046, 147.0651, 129.0548	^#^
					[M-H] ^−^	609.1464	0.4	300.0265, 301.0342, 271.0224, 255.0328, 131.0039	^#^
11	Vitexin	C21H20O10	432.1057	7.06	[M + H] ^+^	433.1126	-0.7	313.0716, 283.0606, 337.0716, 397.0932,415.1026, 367.0819, 379.0819	^#^
					[M-H] ^−^	431.0980	-0.8	311.0561, 283.0614, 341.0668, 353.0693	^#^
12	Isoquercitrin	C21H20O12	464.0955	7.26	[M + H] ^+^	465.1028	-1.7	303.0506, 145.0498, 85.0300	^†^
					[M-H] ^−^	463.0880	-0.5	300.0274, 301.0354, 271.0251, 255.0298,151.0034	^†^
13	Luteolin-7-O-B-D-glucoside (Cynaroside)	C21H20O11	448.1006	7.32	[M + H] ^+^	449.1095	-0.9	449.1095,287.0559	^#^
					[M-H] ^−^	447.0934	0.3	285.0408, 284.0331, 327.0515	^#^
14	3,4-dicaffeoylquinicacid	C25H24O12	516.1268	7.70	[M + H] ^+^	517.1338	-0.5	163.0391,499.1236, 319.0822, 227.0922, 145.0284,135.0443, 355.1038	^†^
					[M-H] ^−^	515.1197	0.3	353.0882, 173.0457, 179.0351, 131.0563, 335.0785,191.0563,335.0785	^†^
15	1,3-dicaffeoylquinicacid	C25H24O12	516.1268	7.86	[M + H] ^+^	517.1338	-0.4	163.0393, 499.1237, 337.0929, 145.0285	^†^
					[M-H] ^−^	515.1195	0.9	353.0880, 191.0562, 179.0352, 161.0250, 335.0782, 135.0459	^†^
16	Isorhamnetin-3-O-galactorhamnoside	C28H32O16	624.1690	7.90	[M + H] ^+^	625.1763	0.4	317.0659, 479.1174	^#^
17	3,5-dicaffeoylquinicacid	C25H24O12	516.1268	7.96	[M + H] ^+^	517.1331	-1.8	163.0388, 499.1215, 319.0818, 337.0812, 135.0437, 145.0280, 355.1084	^†^
					[M-H] ^−^	515.1196	0.9	353.0887, 191.0563, 179.0350, 173.0466, 135.0453, 335.0779	^†^
18	Genistein glucoside	C21H20O10	432.1057	8.24	[M + H] ^+^	433.1127	-0.5	271.0612, 433.1142	^#^
					[M-H] ^−^	431.0981	-0.7	268.0371, 269.0455, 267.0357, 311.0559, 240.0418	^#^
19	4,5-dicaffeoylquinicacid	C25H24O12	516.1268	8.41	[M + H] ^+^	517.1341	0.3	163.0390, 499.1225, 337.0923, 145.0289, 319.0823, 135.0444	^†^
					[M-H] ^−^	515.1195	-0.4	353.0883, 173.0458, 179.0355, 191.0561, 203.0357, 135.0457	^†^
20	Scutellarein	C15H10O6	286.0477	9.32	[M + H] ^+^	287.0553	1.0	287.0553	
					[M-H] ^−^	285.0406	0.6	117.0375, 165.0164, 239.0345	^#^
21	Luteolin	C15H10O6	286.0477	10.37	[M + H] ^+^	287.0553	0.9	153.0184, 241.0487, 137.0217, 135.0435, 161.0234	^#^
					[M-H] ^−^	285.0407	0.9	133.0297, 151.0039, 175.0400, 199.0399, 217.0506, 241.0511	^#^
22	Quercetin	C15H10O7	302.0427	10.39	[M + H] ^+^	303.0500	0.3	303.0517	^†^
					[M-H] ^−^	301.0353	-0.4	151.0032, 178.9977, 121.0303, 273.0414, 107.0148	^†^
23	3-methyl quercetin	C16H12O7	316.0583	11.01	[M + H] ^+^	317.0657	0.5	302.0423, 301.0338, 274.0469, 285.0406, 228.0425	^†^
					[M-H] ^−^	315.0511	0.1	300.0276, 271.0246, 255.0289, 243.0296	^†^
24	apigenin	C15H10O5	270.0528	11.78	[M + H] ^+^	271.0602	0.5	153.0175, 119.0497, 91.0562, 188.9171	^#^
					[M-H] ^−^	269.0455	-0.2	151.0038, 117.0353, 149.0241, 225.0558	^#^
25	arteannuin B	C15H20O3	248.1412	11.89	[M + H] ^+^	249.1484	-0.5	231.1376, 185.1319, 143.0848, 119.0857. 203.1420, 105.0708	Ref.[30]
26	Jaceosidin	C17H14O7	330.0740	12.47	[M + H] ^+^	331.0803	-0.1	316.0571, 301.0337, 273.0393, 245.0438, 168.0056	Ref.[31, 32]
27	Eupatilin	C18H16O7	344.0896	14.61	[M + H] ^+^	345.0964	1.0	330.0737, 329.0778,168.0056, 315.0503	^†^
					[M-H] ^−^	343.0825	0.5	328.0597, 313.0356, 298.0127, 285.0410, 270.0176	^†^
28	Quercetagetin 3,6,7,3'-tetramethyl ether	C19H18O8	374.1002	15.54	[M + H] ^+^	375.1074	-0.1	360.0845, 359.0765, 342.0738,345.0609, 317.0659, 311.0553, 299.0548	^#^
					[M-H] ^−^	373.0931	0.5	358.0703, 343.0466, 300.0580, 315.0515, 328.0230, 285.0043	^#^

^#^ In-house ms/ms library and online database; such as GNPS, MASS bank or Metlin

^†^ Reference standard

## Discussion

Although strategies for IBD treatment have developed, the numbers of patients with IBD are increasing worldwide [33]. However, the development of an effective IBD treatment with few side effects has shown little progress despite continued attempts. Thus, in this study, the potency of *A. argyi* extract was examined to identify possible new therapeutic agents. In a histological analysis of colon tissue of DSS-induced colitis mice, *A. argyi* administration attenuated the disruption of normal colon tissue morphology. Serum cytokines are well known indicators of inflammatory diseases such as IBD, and their expression level correlates with the severity of inflammation [34]. IL-1β, IL-6, and TNF-α are pro-inflammatory cytokines that regulate the Th1 lymphocyte-mediated immune response [35]. Th1 related cytokines were increased in inflamed mucosa from patients with UC and CD, hence inhibiting inflammatory cytokines is a potential treatment avenue [36]. *A. argyi* administration in DSS-induced colitis mice resulted in significant decrease of those cytokines in serum, and expression of inflammation-related genes in colon tissue was also reduced. The scope of the Th1-mediated immune system provoked the development of anti-TNF therapy to attenuate the IBD; however, the sole treatment of anti-TNF agents has shown partial triumph for patients [37]. We found broad anti-inflammation effects of *A. argyi*, including decreases of multiple cytokines, prostaglandin, and chemokines in serum and colon lesion from colitis mice. In addition, the decreased MPO, an indicator of neutrophils and macrophages, also reinforced our conclusion that *A. argyi* treatment ameliorates of inflammation.

NF-κB, a major transcriptional factor involved in immune and inflammatory responses, contributes to pathogenesis of various inflammatory diseases such as IBD, rheumatoid arthritis, and atherosclerosis [38]. In our study, *A. argyi* treatment highly inhibited translocation of NF-κB into cell nuclei in vitro, and activation of NF-κB in vivo. As NF-κB is a key regulator of inflammatory genes, there is ample evidence implicating it in IBD pathogenesis [39]; therefore, we monitored the inhibition of NF-κB by *A. argyi* treatment as well as its possible causes. Previous studies have proposed cross-talk between NF-κB and Nrf2, a central regulator of phase 2 detoxification [40]. HO-1, regulated by Nrf2, is an enzyme that catalyzes decomposition of heme molecules. Carbon monoxide, produced by the catalysis of HO-1, inhibits NF-κB, along with the expression of several chemokines such as IL-6 and TNF-α secreted by the activation of NF-κB [41]. Rutaecarpine and carnosic acid ameliorated inflammatory responses in DSS-induced colitis mice via Nrf2 activation by interfering with Keap1-Nrf2 binding [42, 43]; such attempts have shown promise as emerging therapeutic strategies for IBD. TLR4 has been suggested as an intermediary between NF-κB and Nrf2, and Maresin-1 (pro-resolving lipid synthesized in macrophages) reduced TLR4/NF-κB mechanism and activated Nrf2 in DSS-induced colitis mice [44]. Likewise, A. argyi not only decreased the NF-κB signaling pathway but also induced Nrf2 activation in LPS-induced macrophage cells.

Although the defense mechanism against IBD is mainly regulated by colon intestinal epithelial cells [45], previous studies have revealed that some gut-associated lymphoreticular tissues (GALTs), remotely located from colon like mesenteric lymph nodes and PPs, could affect the severity of colitis [46, 47]. PP is representative of GALT in that it is mainly located in luminal surfaces on the intestine but infrequently on colon tissue as well, and strongly contributes to immune responses between host and pathogens through recognizing and presenting of foreign antigens using abundant DCs [48]. PPs are mucosal immune barriers with front-line function against enterobacteria, and they aid CD repression by regulating Th2-type T cell responses [49]. Meanwhile, we also focused on the spleen due to its role in reticuloendothelial system and its versatile functions in immune responses [50]. Severe IBD sometimes invades the spleen, causing abscesses [51], while the spleen-associated central immune system may attenuate colitis by promoting CD11b^+^Gr1^+^ myeloid cells and activating the cholinergic anti-inflammatory pathway [52, 53]. However, the importance and role of both PPs and spleen in IBD have been neglected.

We observed significantly elevated inflammation in both PPs and spleens in DSS-induced colitis mice. Especially in splenic tissue, the DSS-induced IBD caused an increase in F4/80^+^CD11c^+^ macrophage infiltration and partially disrupted microstructures in splenic tissue. *A. argyi* treatment effectively ameliorated inflammatory responses in both lymphoid tissues as observed in colon tissue. This suggests that *A. argyi* treatment may help to relieve inflammation in the whole body due to its effect on the immune defense system in lymphoid tissues. With both mitigation of aggressive inflammatory cytokines in the PPs and the spleen after *A. argyi* treatment, we detected significant increases in *IL-10* gene expression in both tissues, suggesting the possibility that *A. argyi* reinforces immunomodulatory effects in IBD pathogenesis, with the activation of IL-10 importance to the mechanism. IL-10 is a central immunomodulatory cytokine that suppresses pro-inflammatory responses in both innate and adaptive immune system, and improves lesions caused by infections and inflammation [54]. The protective function of IL-10 in the progress of IBD has been investigated; IL-10 broadly affects the innate and adaptive immune systems. The progress of IBD pathogenesis loosens the epithelial barrier of the bowel, allowing the intrusion of enteric bacteria, which is thought to trigger IBD. IL-10 helps to rapidly eliminate pathogenic microbes by stimulating the innate immune defense system of the host, and efficiently mitigates IBD development [54]. The role of IL-10 in IBD pathogenesis was also demonstrated using *Il10*^−/−^ mice; it increased infiltration of leukocyte and macrophages with persistent colitis symptoms compare to normal-type mice [55]. The relationship between IL-10 and IBD pathology has been previously reported [56], as well as the identification of a polymorphism of *IL-10* that increases susceptibility of IBD in Spain [57]. Consequently, IL-10 associated immunomodulatory reactions would be activated in lymphoid tissues by *A. argyi* treatment, and that such treatment would assist the attenuation of inflammation in colitis, although this postulate requires further confirmation.

Due to the advantages of fewer side effects and increased accessibility relative to conventional drugs, beneficial effects of medicinal herbs and functional foods have been studied [58]; in addition, herbal therapeutics show multi-targeting effectiveness derived from their complex chemical constituents. *A. argyi* is a well-known traditional dietary plant with anti-inflammatory and anti-oxidative properties [59]. *A. argyi* ethanol extracts evidently relieved IBD both at the histological and molecular levels; however, the exact chemical constituents remain relatively unknown. We illustrated the anti-inflammatory effects of some major chemical compounds found in *A. argyi* extract: two characteristic flavones of *A. argyi* (eupatilin and jaceosidin) and three kinds of isomers of DCQA family compounds (chlorogenic acid, 3,5-DCQA, and 4,5-DCQA) are candidates, as they showed strong activity, although their respective effectiveness were much lower than that of *A. argyi* extract. In addition, several other chemical constituents of *A. argyi* extract have also been reported to have anti-inflammatory effects [19]. For example, scopoletin was shown to exert anti-inflammatory effects in animal models of various inflammation-associated disorders [60, 61]; esculetin [62], rutin [63], and vitexin [64] also affect inflammation. This may indicate that the exceptional effectiveness of *A. argyi* extract is attributed to the simultaneous activities of multiple chemical compounds. Bao et al. [65] suggested that even the polysaccharide fraction of *A. argyi* may have immunomodulatory function, though these polysaccharides may not have been contained in our extract due to differences in extraction procedures.

## Conclusions

We determined whether the activity of *A. argyi* ethanol extract can alleviate DSS-induced colitis symptoms and its mechanism. In a mouse model where we induced acute colitis via treatment with a 3% DSS solution for ten days, we found that administration of *A. argyi* eased colitis-induced histological changes in colon tissue and decreased pro-inflammatory cytokine levels in serum. We also found that *A. argyi* ameliorates NF-κB activation in colitis mice and impedes its translocation into nuclear using in vitro model, and downregulated inflammatory genes. We also examined the effect of *A. argyi* in PPs and spleen tissue and found significant improvements in inflammation in both tissues. In addition, the immunomodulatory action of *A. argyi* may be attributed to the upregulation of IL-10. A total of 28 chemical compounds were identified in the UPLC-MS/MS analysis, with several bioactive compounds with anti-inflammatory properties, including unique flavones (eupatilin and jaceosidin). These bioactive compounds contributed to the exceptional potency of *A. argyi* extract identified in the study, suggesting its potential therapeutic use against IBD.

## Supplementary Information

**Additional file 1:** **Table S1. **Sequences of qRT-PCR primers used in the study.

**Additional file 2:** **Figure S1. **MS/MS spectral patterns of each chemical compound identified in the *A.argyi *ethanol extract.

**Additional file 3:** **Figure S2. **NO assay results of certain major constituents (3,5-DCQA, 4,5-DCQA, chlorogenic acid, eupatilin, and jaceosidin) found in the *A.argyi* ethanol extract.

**Additional file 4.**

## Data Availability

Not applicable.

## Abbreviations

IBD: Inflammatory bowel disease
UC: Ulcerative colitis
CD: Crohn’s disease
TLR: Toll-like receptor
DC: Dendritic cells
PP: Peyer’s patch
5-ASA: 5-Aminosalicylates
DSS: Dextran sodium sulfate
MEM: Minimum essential media
NO: Nitric oxide
LPS: Lipopolysaccharide
CMC: Carboxymethyl cellulose solution
DAI: Disease activity index
H&E: Hematoxylin and eosin
ELISA: Enzyme-linked immunosorbent assay
IL: Interleukin
TNF-α: Tumor necrosis factor-α
MPO: Myeloperoxidase
PGE_2_: Prostaglandin E_2_
AST: Aminotransferase
ALT: Alanine aminotransferase
Cox2: Cyclooxygenase-2
HO-1: Heme oxygenase-1
Nrf2: Nuclear factor-erythroid factor 2-related factor 2
NF-κB: Nuclear factor-κB
iNOS: Inducible nitric oxide synthase
qRT-PCR: Quantitative real-time polymerase chain reaction
PBS: Phosphate-buffered saline
BSA: Bovine serum albumin
UPLC-QTOF-MS/MS: Ultra-high performance liquid chromatography-quadrupole time-of-flight tandem mass spectrometry
ESI: Electrospray ionization
SEM: Standard error of the mean
ANOVA: Analysis of variance
ICAM-1: Intercellular adhesion molecule 1
MCP1: Monocyte chemoattractant protein-1
DCQA: Dicaffeoylquinic acid
GALT: Gut-associated lymphoreticular tissue
