# Supplementary material for: Artemisia argyi extract alleviates inflammation in a DSS-induced colitis mouse model and enhances immunomodulatory effects in lymphoid tissues
Source: BMC Complement Med Ther. 2022 Mar 11;22:64. doi: 10.1186/s12906-022-03536-x (PMC8917695; doi:10.1186/s12906-022-03536-x)
Supplement: Supplementary file 1 — Additional file 1: Table S1. Sequences of qRT-PCR primers used in the study. [file 12906_2022_3536_MOESM1_ESM.docx]

Table S1. Sequences of qRT-PCR primers used in the study.

| **Gene** | **Primer** | **Sequence** |
| --- | --- | --- |
| Mouse *IL-1β* | Forward | 5′-GGT ACA TCA GCA CCT CAC AA-3′ |
|  | Reverse | 5′-TTA GAA ACA GTC CAG CCC ATA C-3′ |
| Mouse *IL-6* | Forward | 5′-CCC AAC AGA CCT GTC TAT ACC -3′ |
|  | Reverse | 5′-CAG CTT ATC TGT TAG GAG AGC-3′ |
| Mouse *TNFα* | Forward | 5′-TCC CCA AAG GGA TGA GAA GTT C-3′ |
|  | Reverse | 5′-GGG AGT AGA CAA GGT ACA AC-3′ |
| Mouse *ICAM-1* | Forward | 5′-GCC TTG GTA GAG GTG ACT GAG-3′ |
|  | Reverse | 5′-GAC CGG AGC TGA AAA GTT GTA-3′ |
| Mouse *MCP-1* | Forward | 5′-AGG TGT CCC AAA GAA GCT GTA-3′ |
|  | Reverse | 5′-ATG TCT GGA CCC ATT CCT TCT-3′ |
| Mouse *iNOS* | Forward | 5′-CCA AGC CCT CAC CTA CTT CC-3′ |
|  | Reverse | 5′-CTC TGA GGG CTG ACA CAA GG-3′ |
| Mouse *IL-10* | Forward | 5′-CAG AGC CAC ATG CTC CTA GA-3′ |
|  | Reverse | 5′-GGC AAC CCA AGT AAC CCT TA-3′ |
| Mouse *F4/80* | Forward | 5′-CTT TGG CTA TGG GCT TCC AGT-3′ |
|  | Reverse | 5′-GCA AGG ACA GAG TTT ATC GTG-3′ |
| Mouse *CD11c* | Forward | 5′-CTG GAT AGC CTT TCT GCT G-3′ |
|  | Reverse | 5′-GCA CAC TGT GTC CGA ACT C-3′ |
| Mouse *GAPDH* | Forward | 5′-GCC CAA TAC GAC CAA ATC C-3′ |
|  | Reverse | 5′-AGC CAC ATC GCT CAG ACA C-3′ |
