# Supplementary material for: Artemisia argyi extract alleviates inflammation in a DSS-induced colitis mouse model and enhances immunomodulatory effects in lymphoid tissues
Source: BMC Complement Med Ther. 2022 Mar 11;22:64. doi: 10.1186/s12906-022-03536-x (PMC8917695; doi:10.1186/s12906-022-03536-x)
Supplement: Supplementary file 2 — Additional file 2: Figure S1. MS/MS spectral patterns of each chemical compound identified in the A.argyi ethanol extract. [file 12906_2022_3536_MOESM2_ESM.docx]

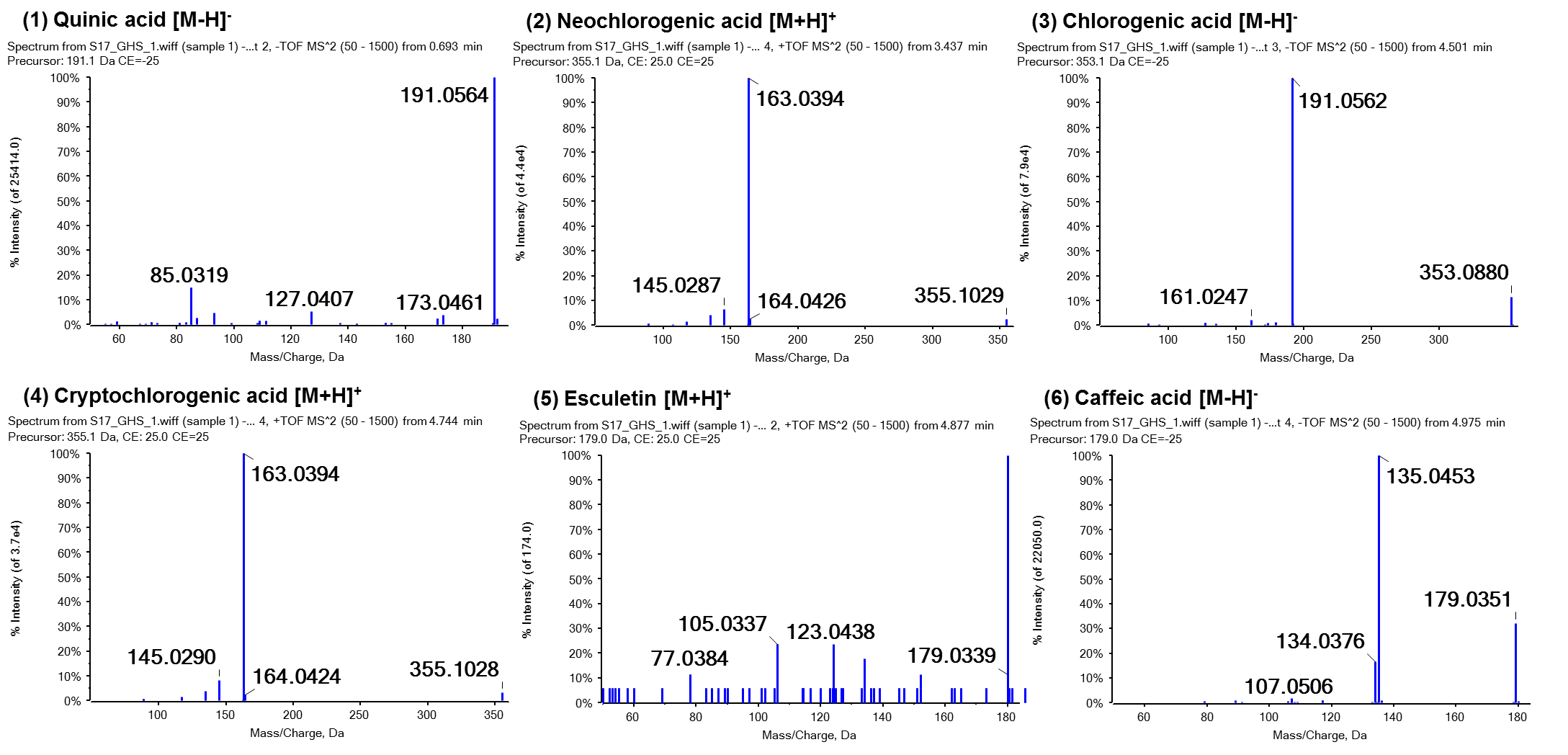


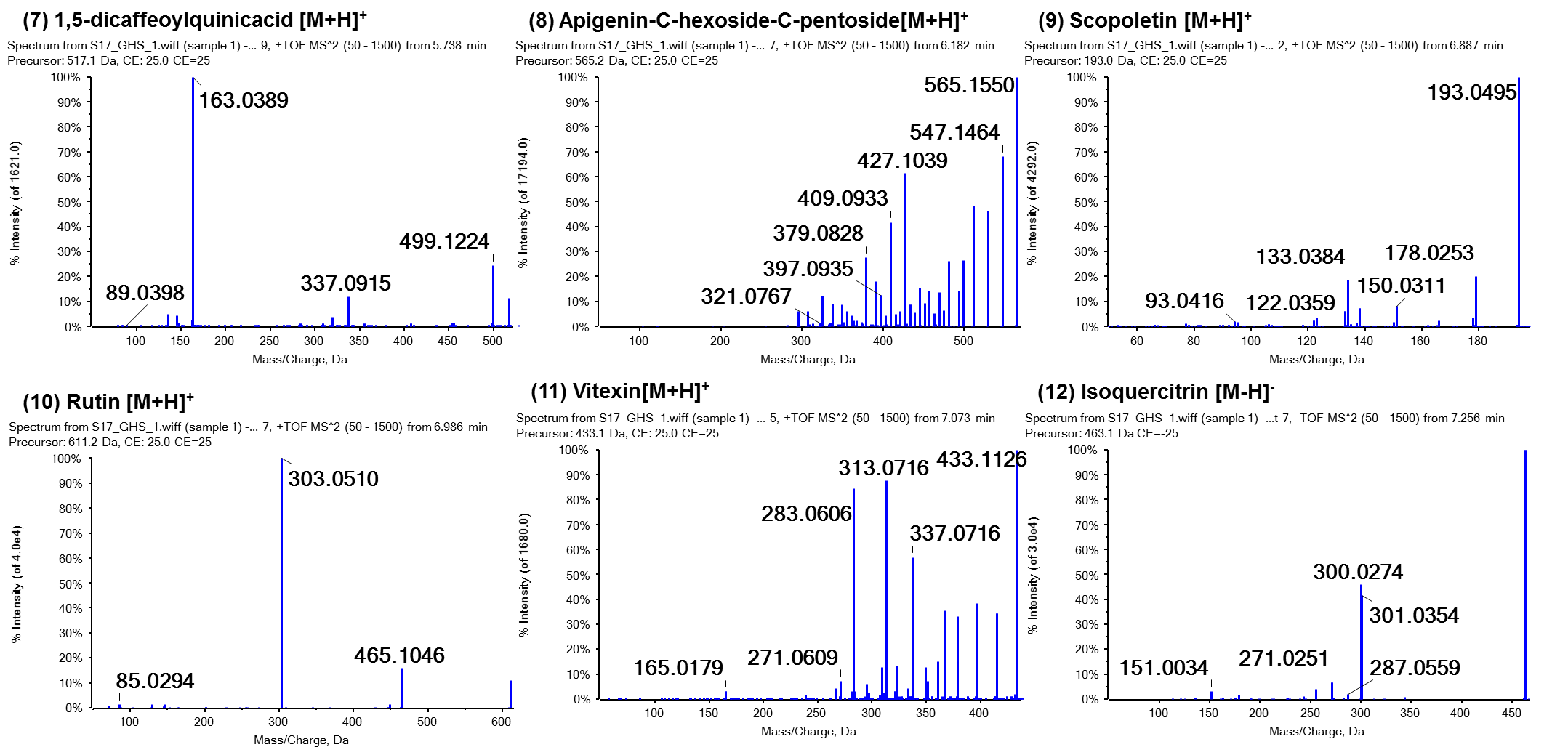


**Figure S1.** **MS/MS spectral patterns of each chemical compound identified in the *A. argyi* ethanol extract.**


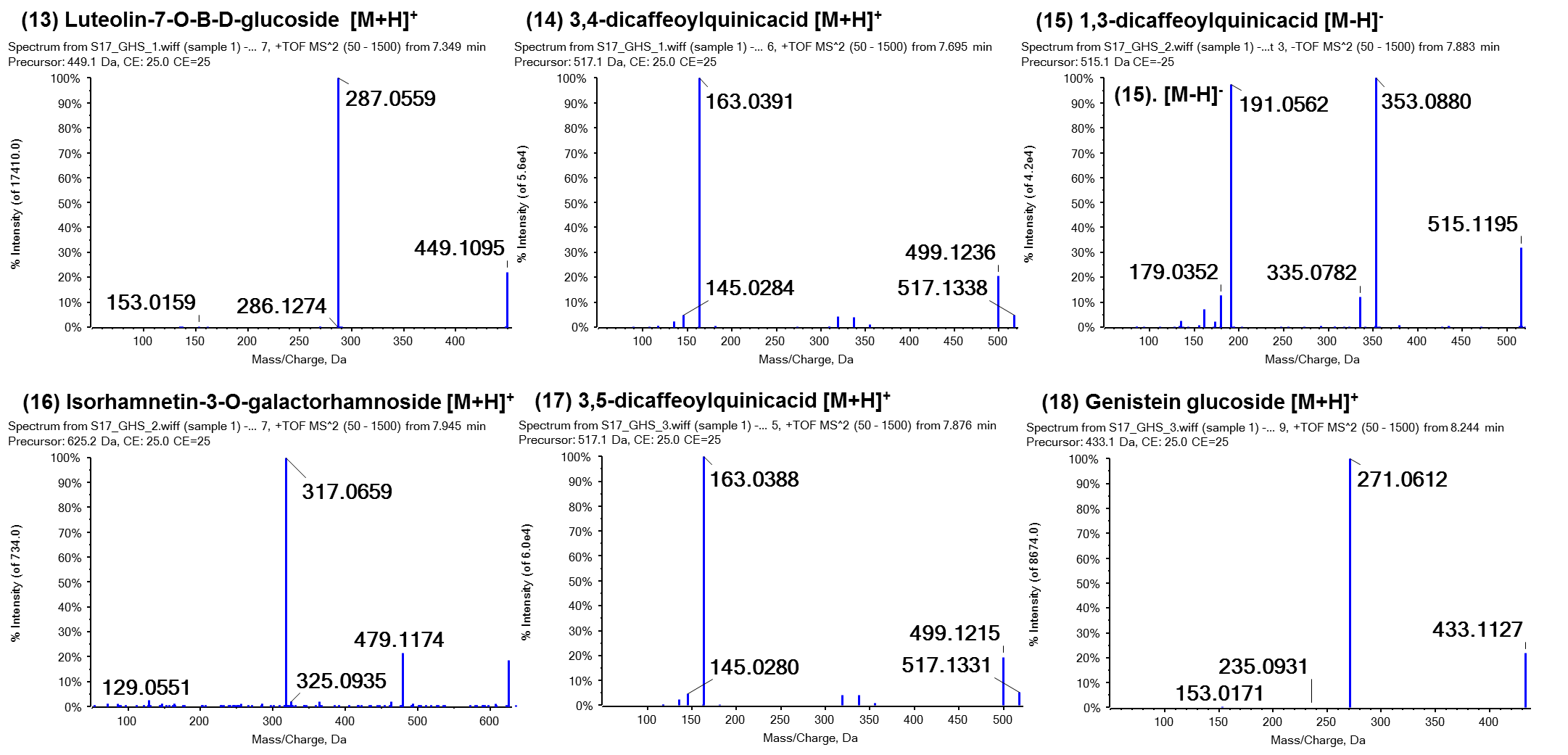


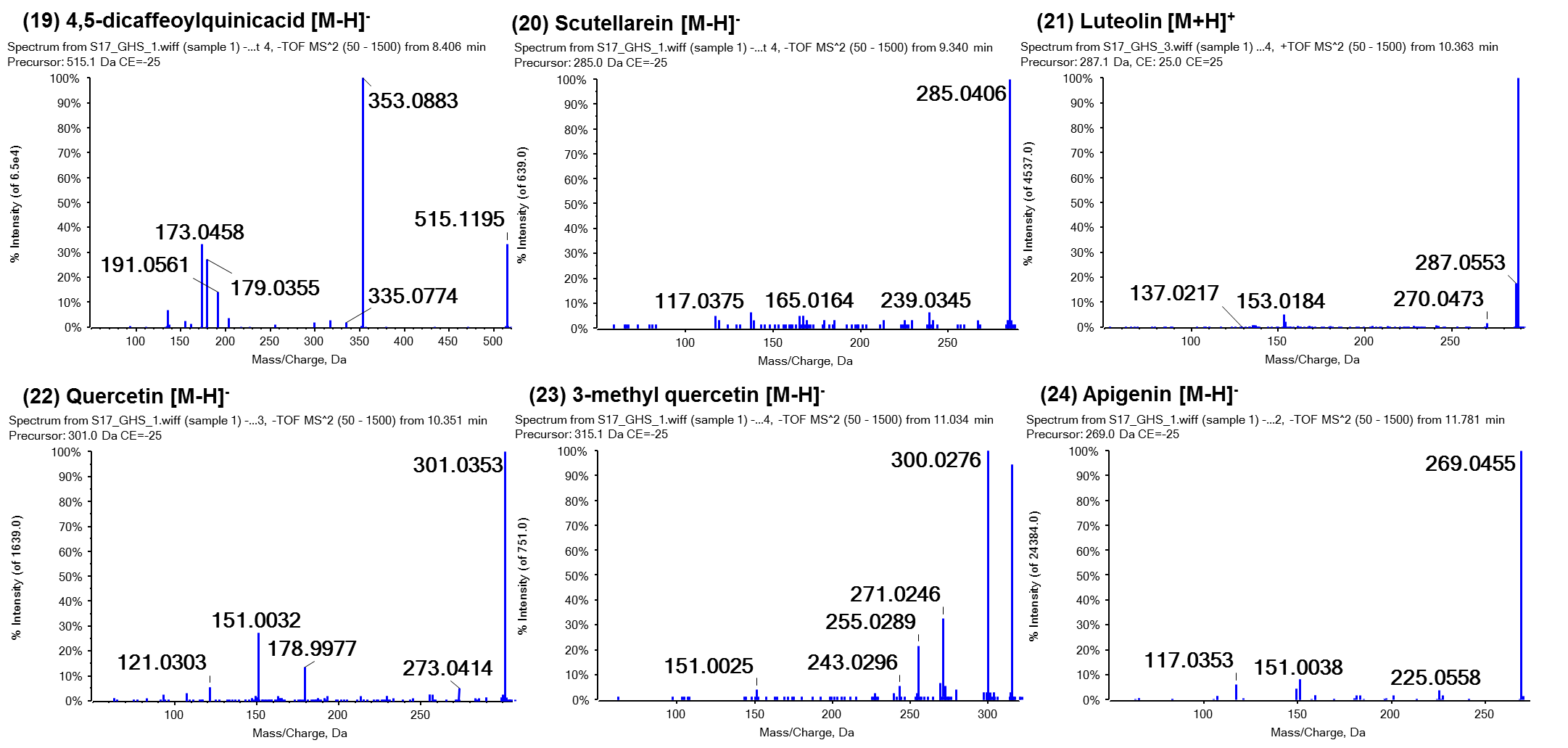


**Figure S1 (continued). MS/MS spectral patterns of each chemical compound identified in the *A. argyi* ethanol extract.**


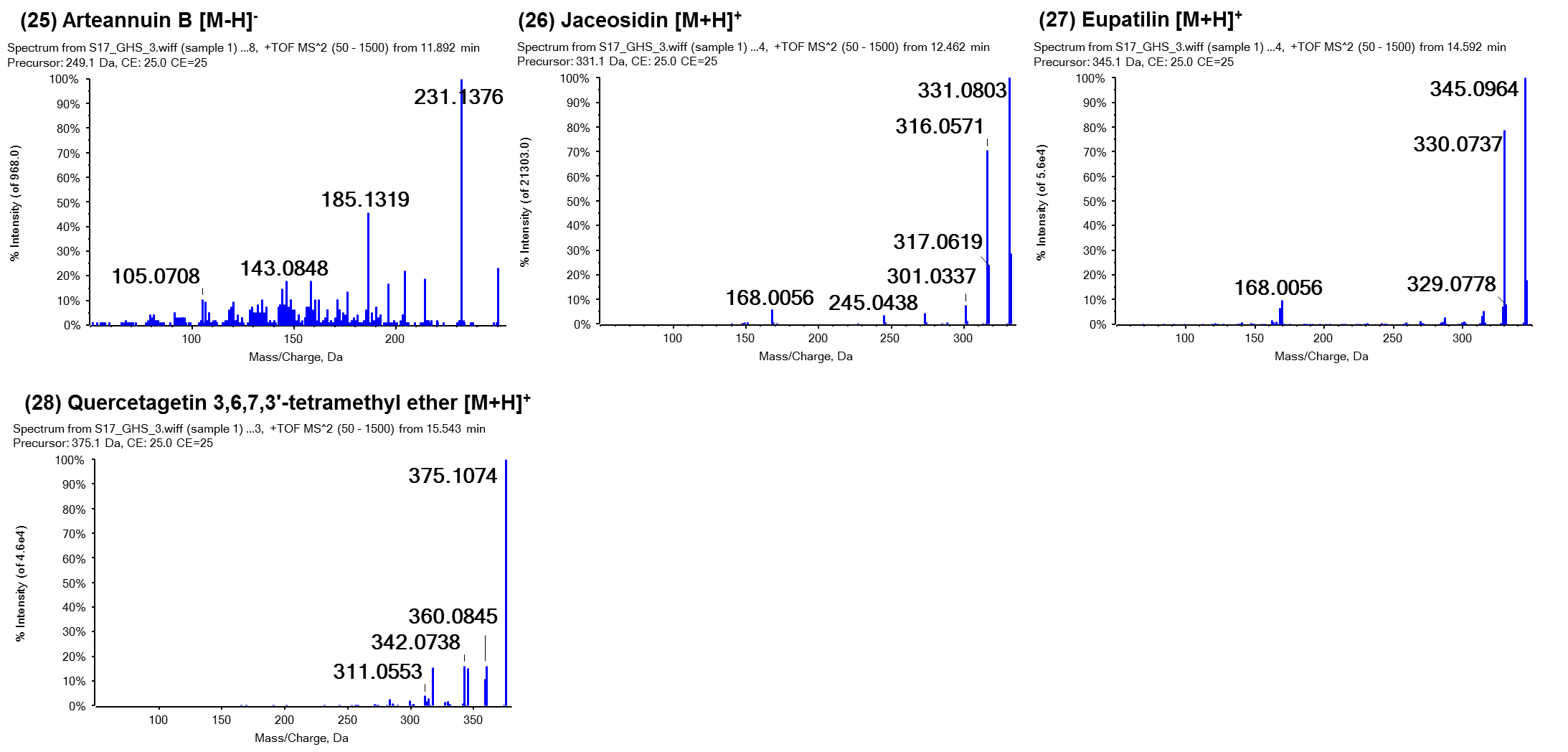


**Figure S1 (continued). MS/MS spectral patterns of each chemical compound identified in the *A. argyi* ethanol extract.**
