# Supplementary material for: Artemisia argyi extract alleviates inflammation in a DSS-induced colitis mouse model and enhances immunomodulatory effects in lymphoid tissues
Source: BMC Complement Med Ther. 2022 Mar 11;22:64. doi: 10.1186/s12906-022-03536-x (PMC8917695; doi:10.1186/s12906-022-03536-x)
Supplement: Supplementary file 3 — Additional file 3: Figure S2. NO assay results of certain major constituents (3,5-DCQA, 4,5-DCQA, chlorogenic acid, eupatilin, and jaceosidin) found in the A.argyi ethanol extract. [file 12906_2022_3536_MOESM3_ESM.docx]

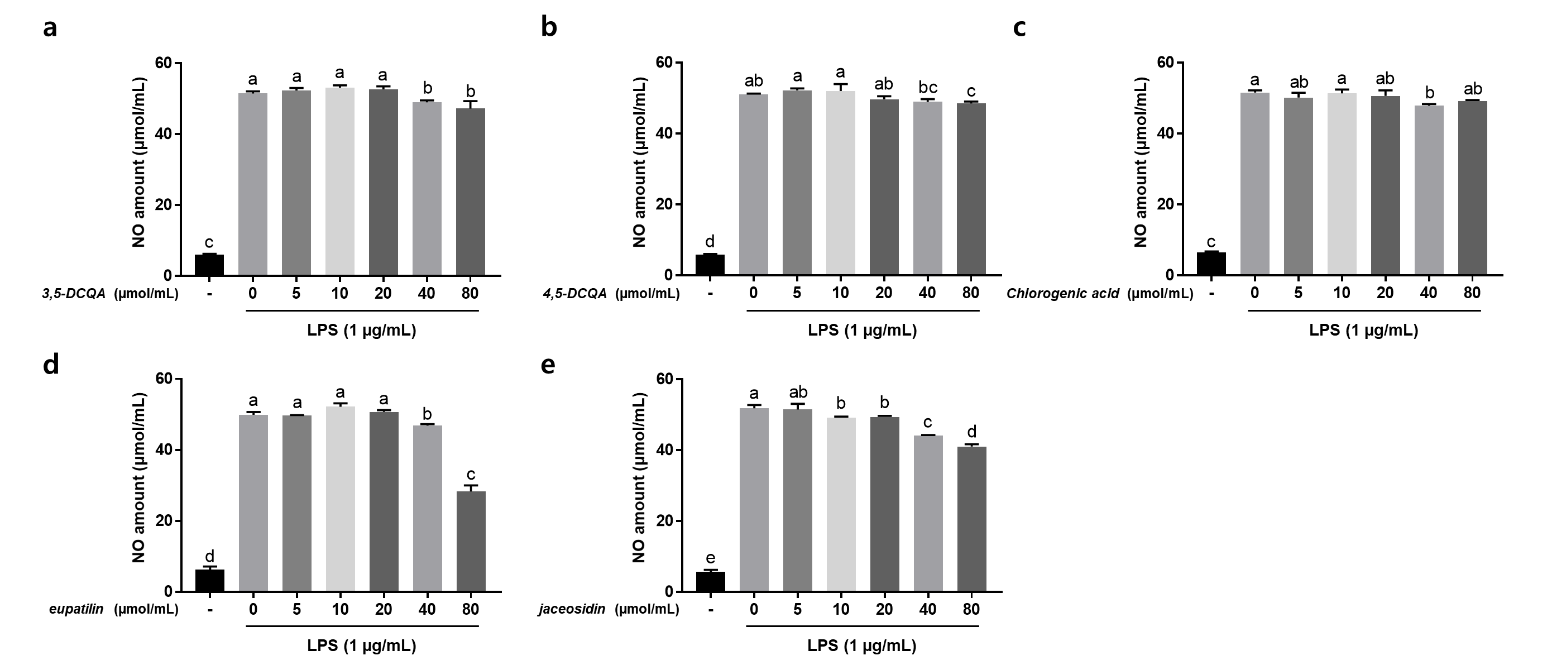


Figure S2. NO assay results of certain major constituents (3,5-DCQA, 4,5-DCQA, chlorogenic acid, eupatilin, and jaceosidin) found in the *A. argyi* ethanol extract.
