# Supplementary material for: Artemisia argyi extract alleviates inflammation in a DSS-induced colitis mouse model and enhances immunomodulatory effects in lymphoid tissues
Source: BMC Complement Med Ther. 2022 Mar 11;22:64. doi: 10.1186/s12906-022-03536-x (PMC8917695; doi:10.1186/s12906-022-03536-x)

**Images of uncropped gels**

Cox2 (Fig. 1c)


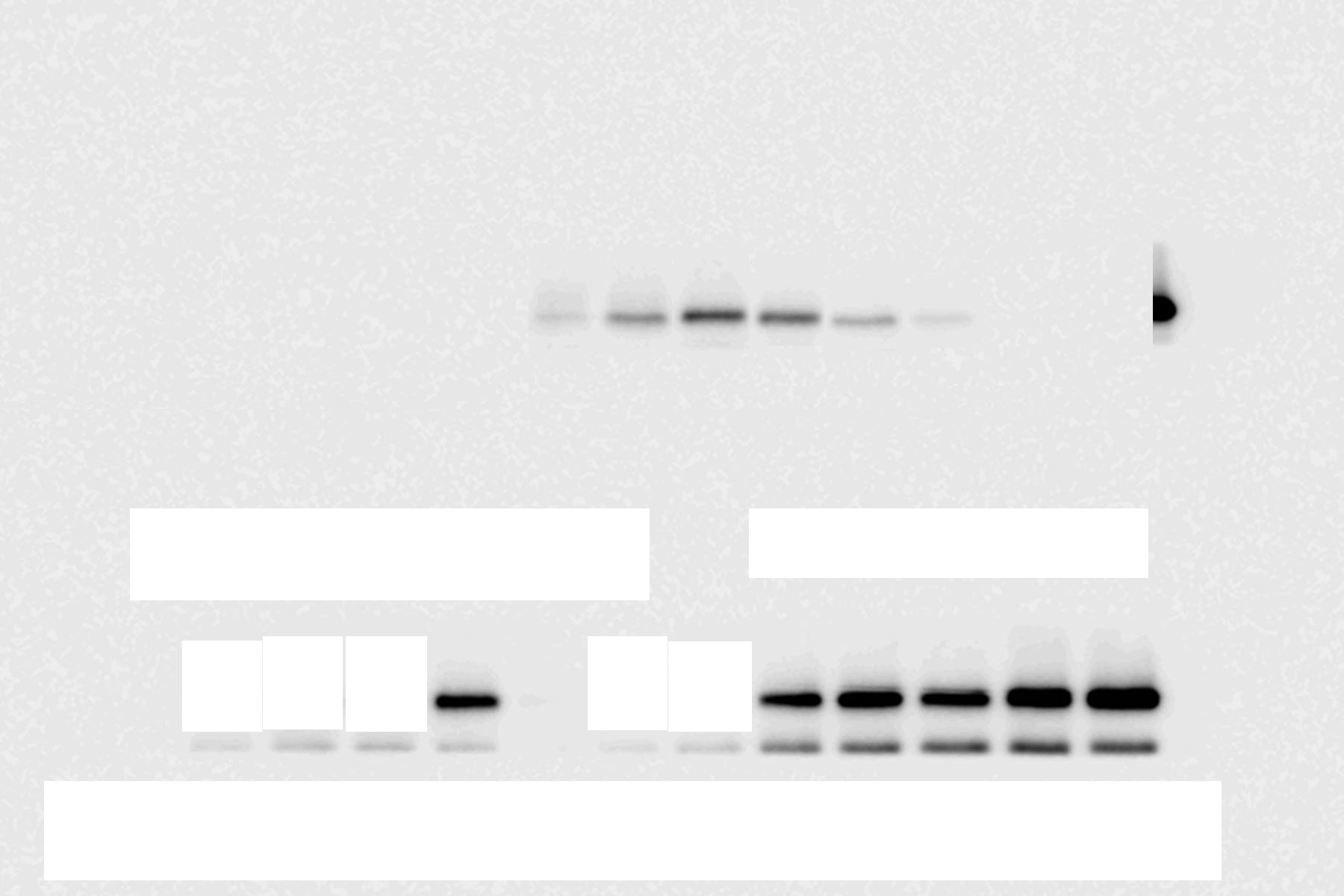


iNOS (Fig. 1c)


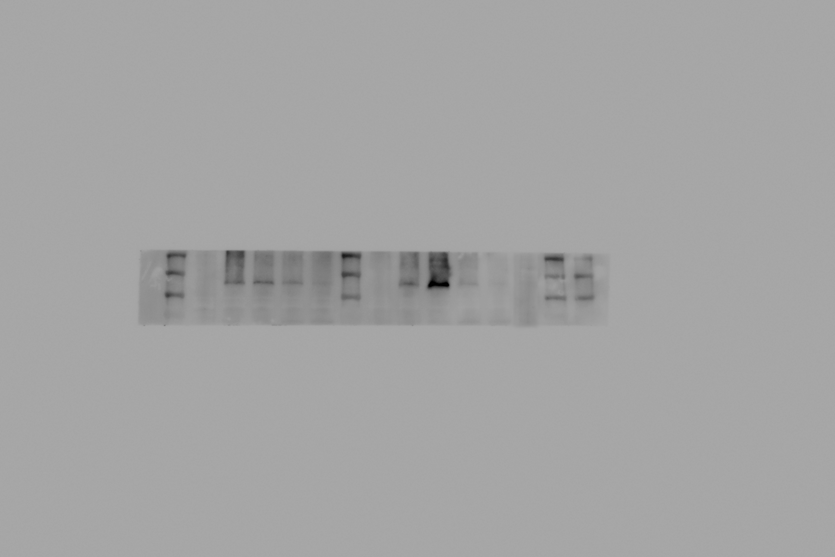


β-actin (Fig. 1c)


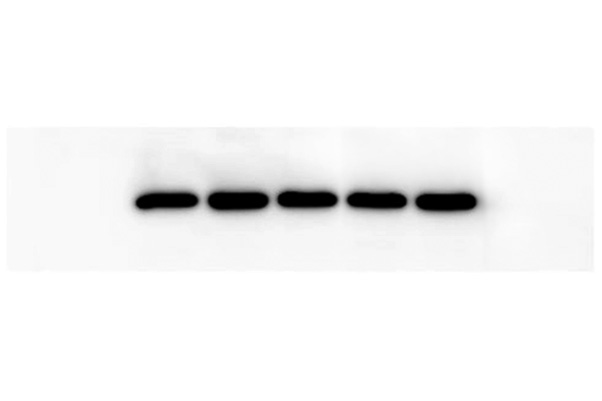


LaminB (Nuclear fraction) (Fig. 1d)


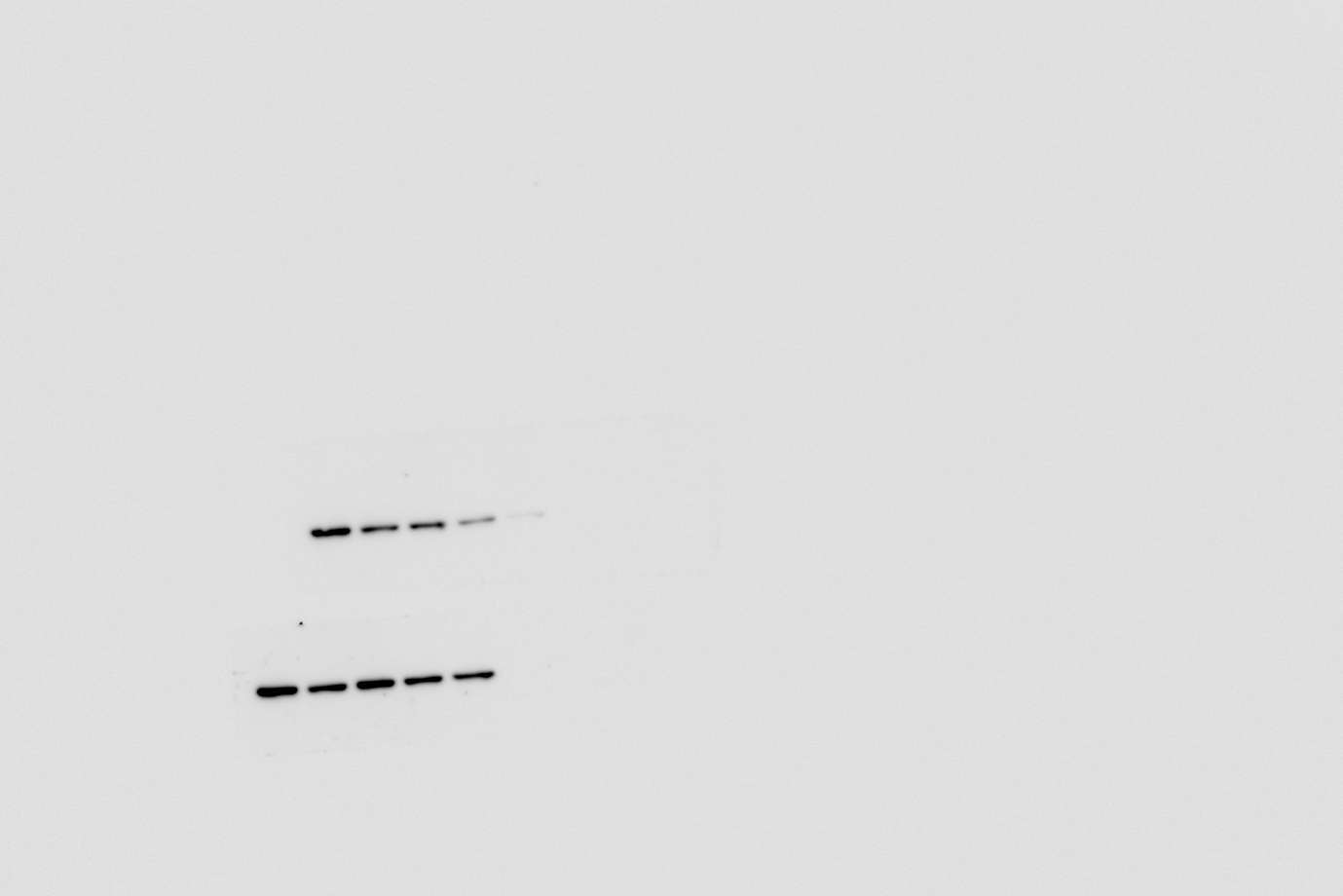


NF-κB (nuclear fraction) (Fig. 1d)


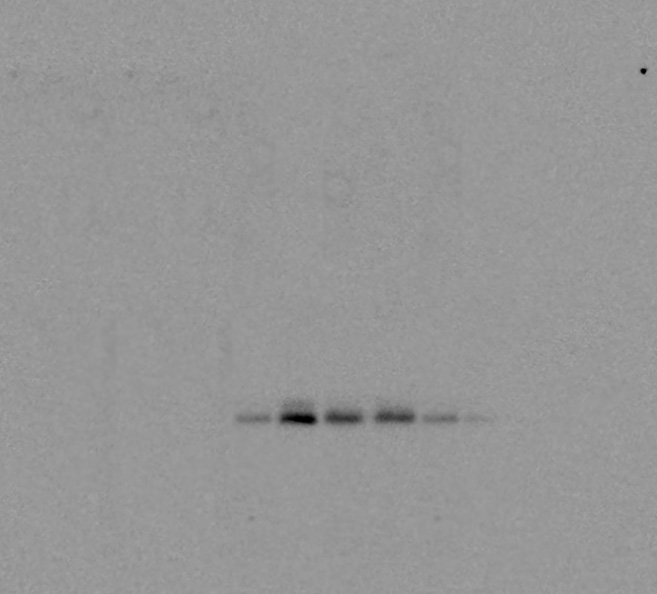


NF-κB (cytosolic fraction) (Fig. 1d)


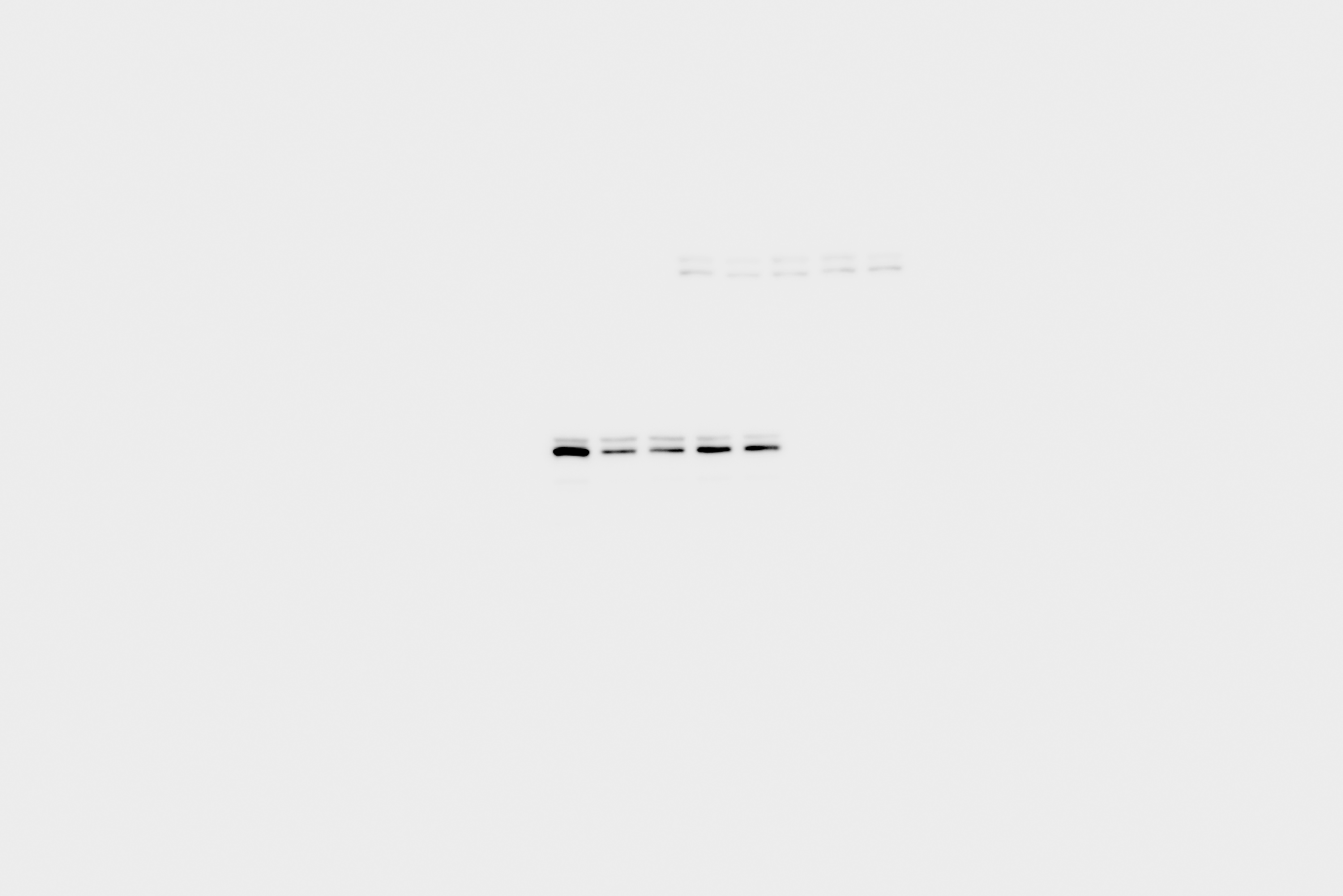


IκBα (cytosolic fraction) (Fig. 1d)


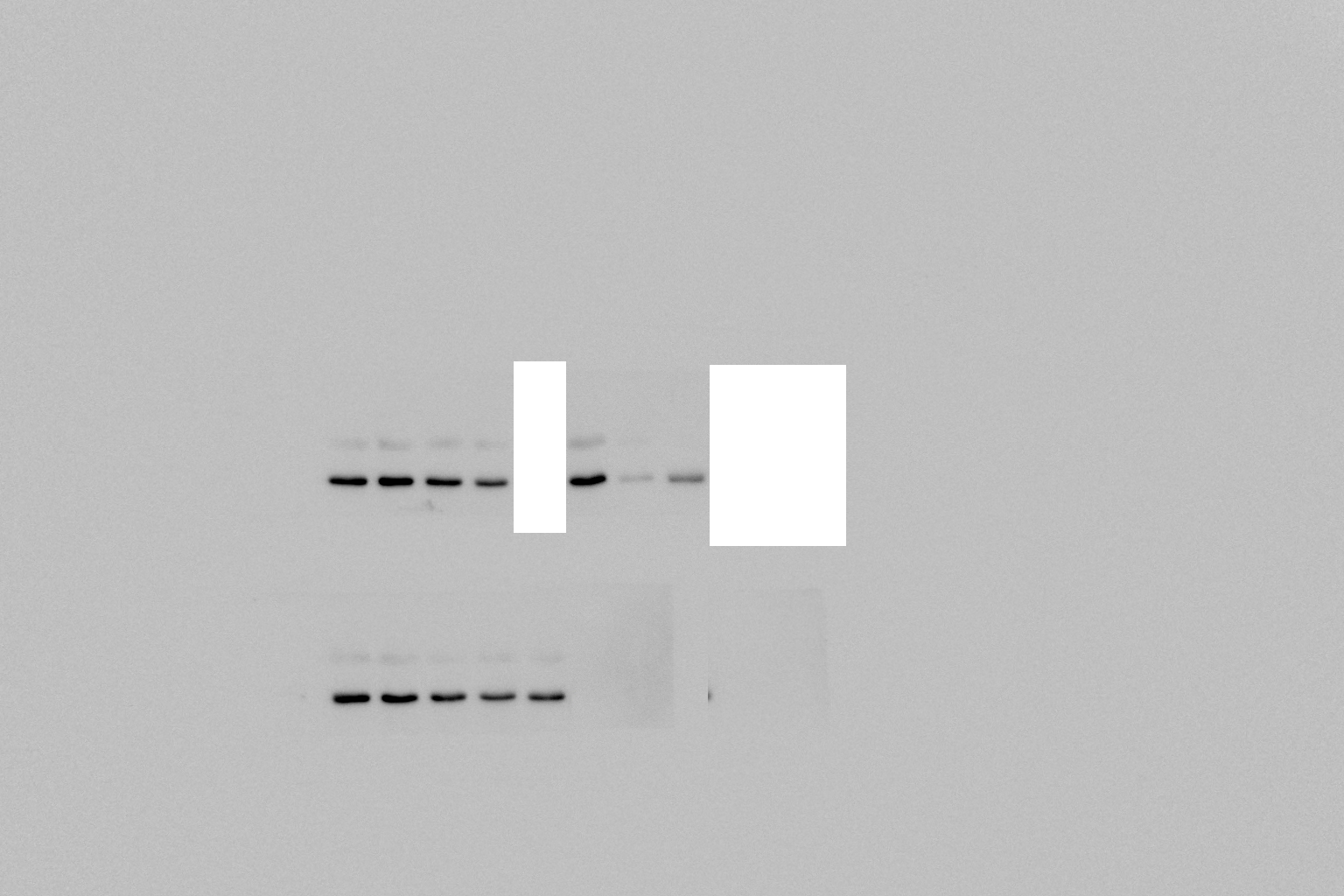


β-actin (cytosolic fraction) (Fig. 1d)


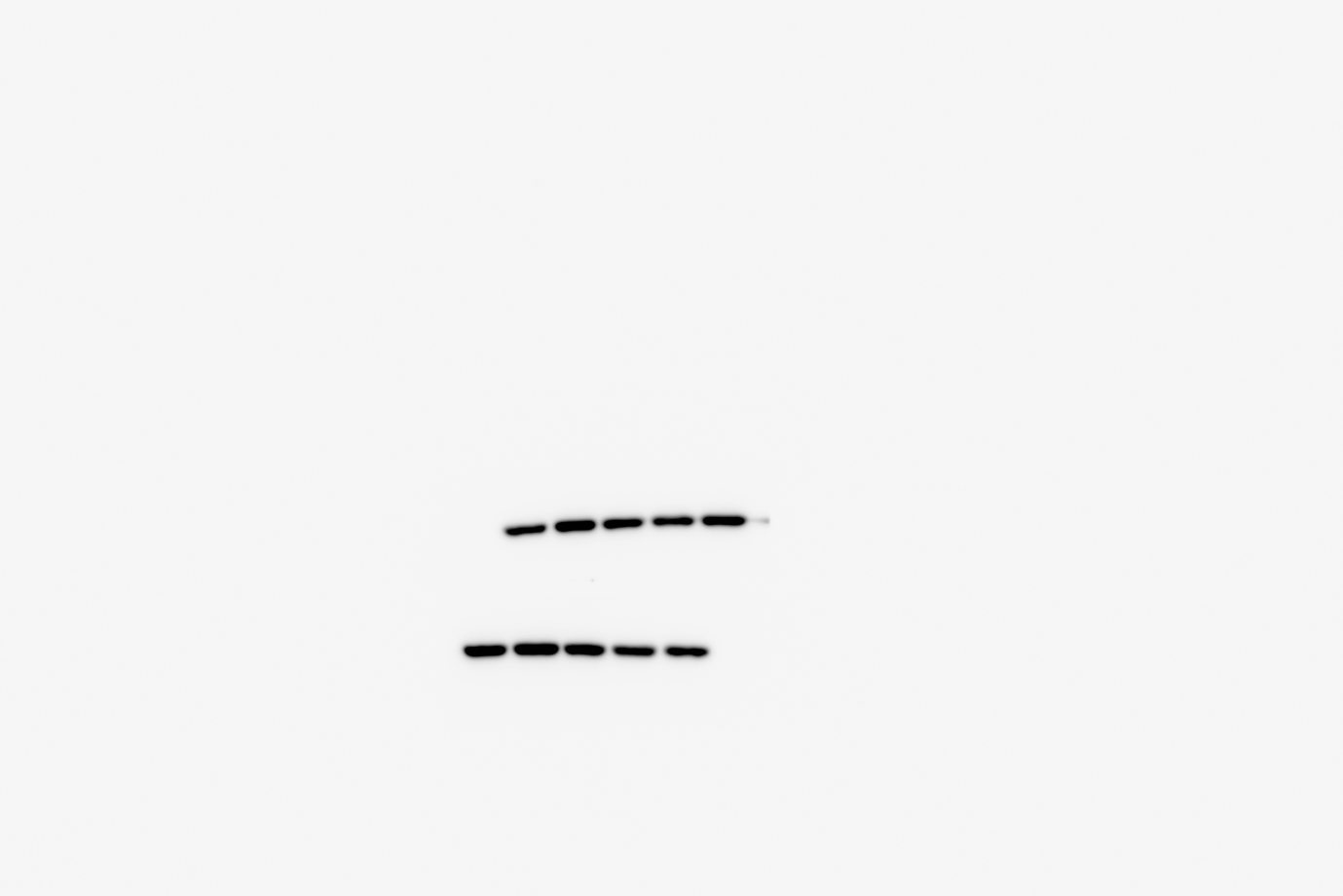


Nrf2 (Fig. 1e)


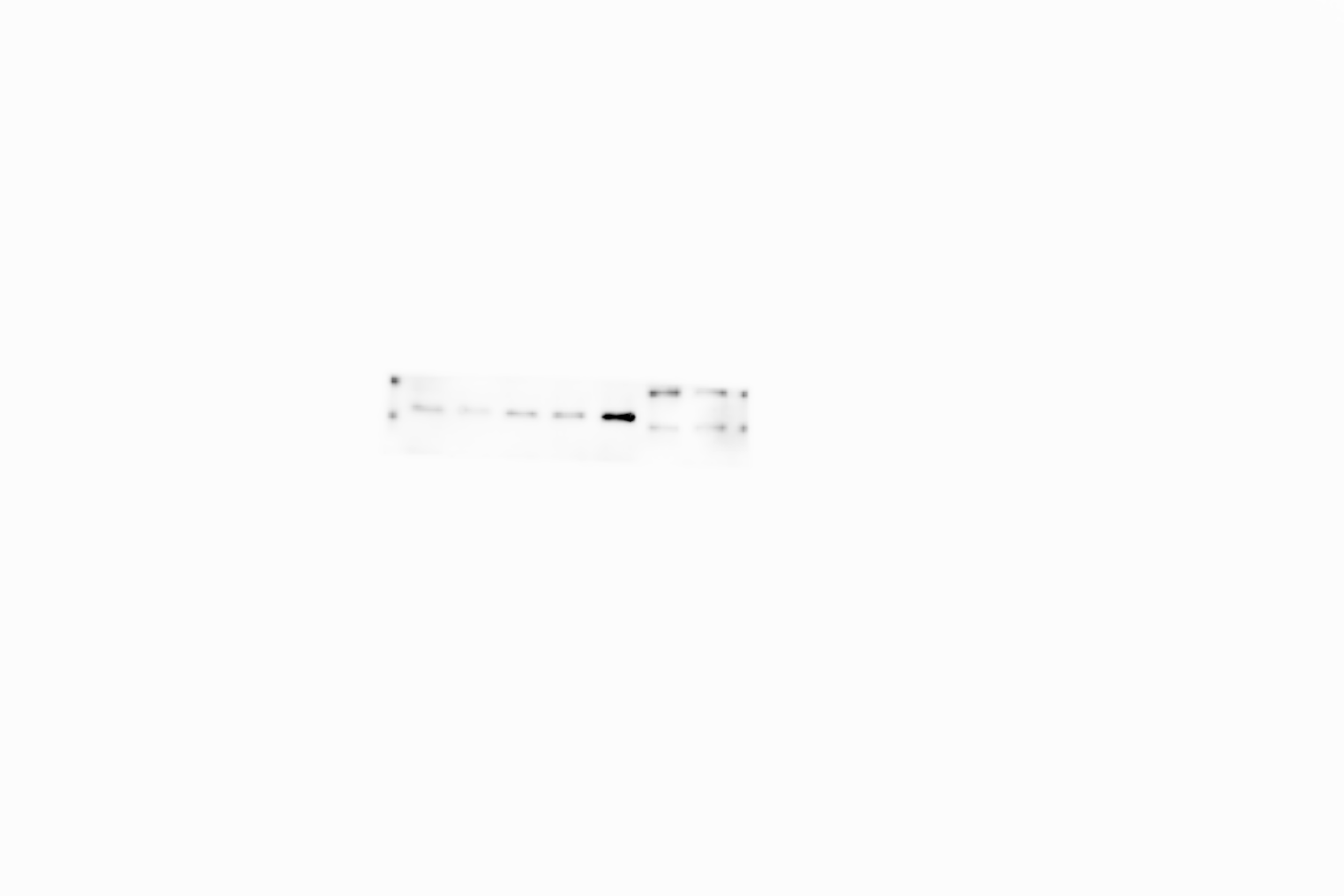


80 kDa

100 kDa

HO-1 (Fig. 1e)


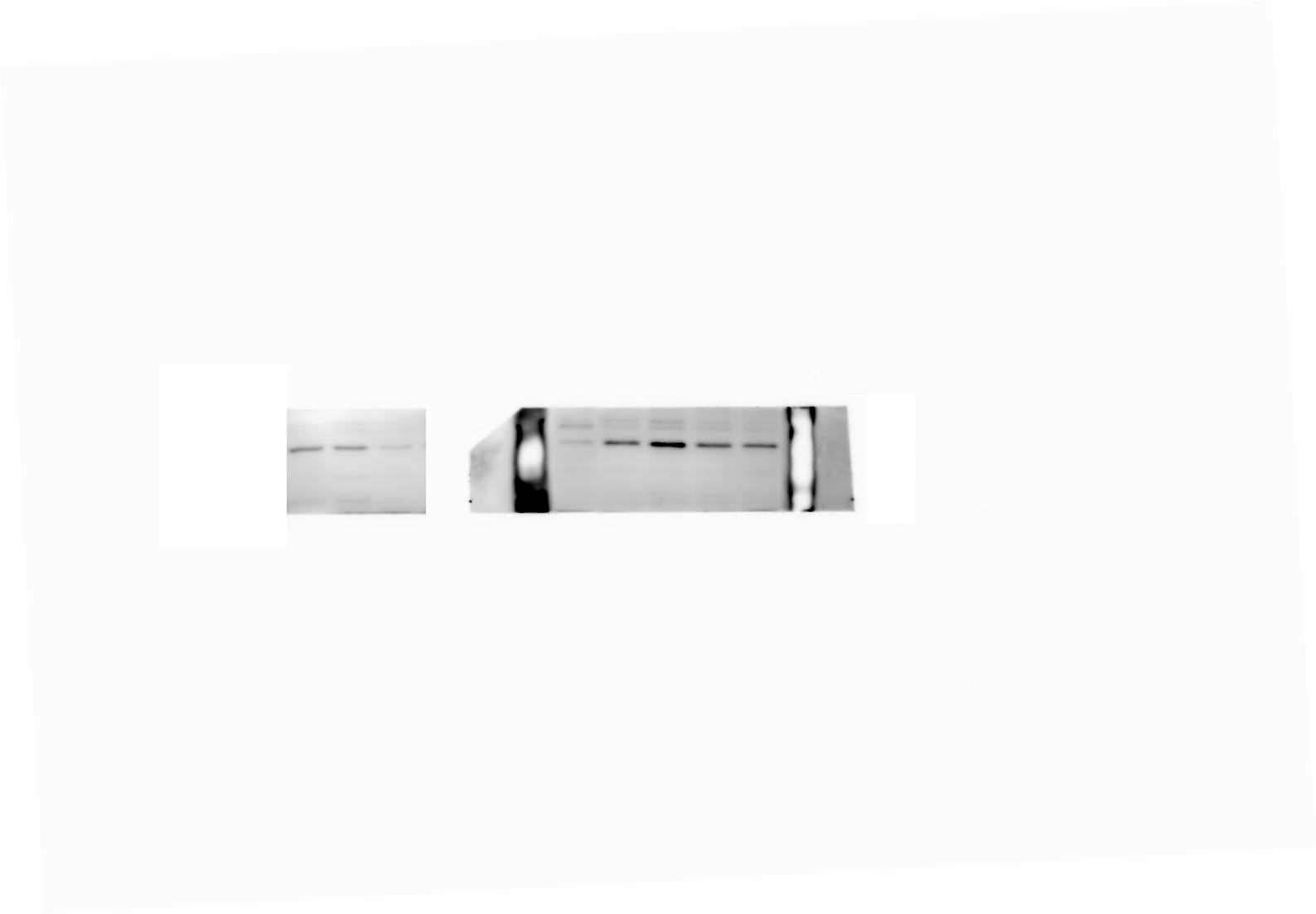


β-actin (cytosolic fraction) (Fig. 1e)


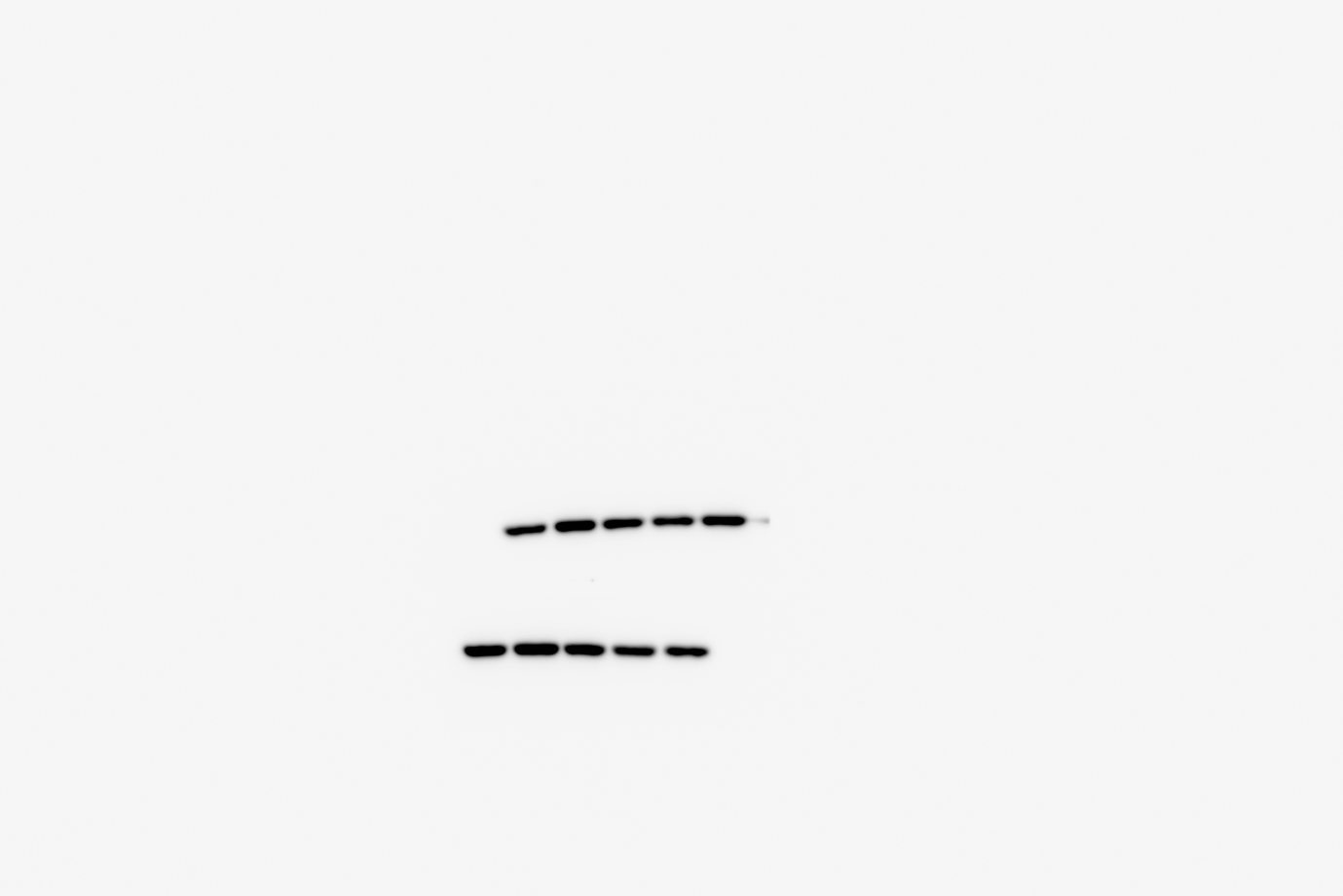


Nrf2 (Nuclear fraction) (Fig. 1f)


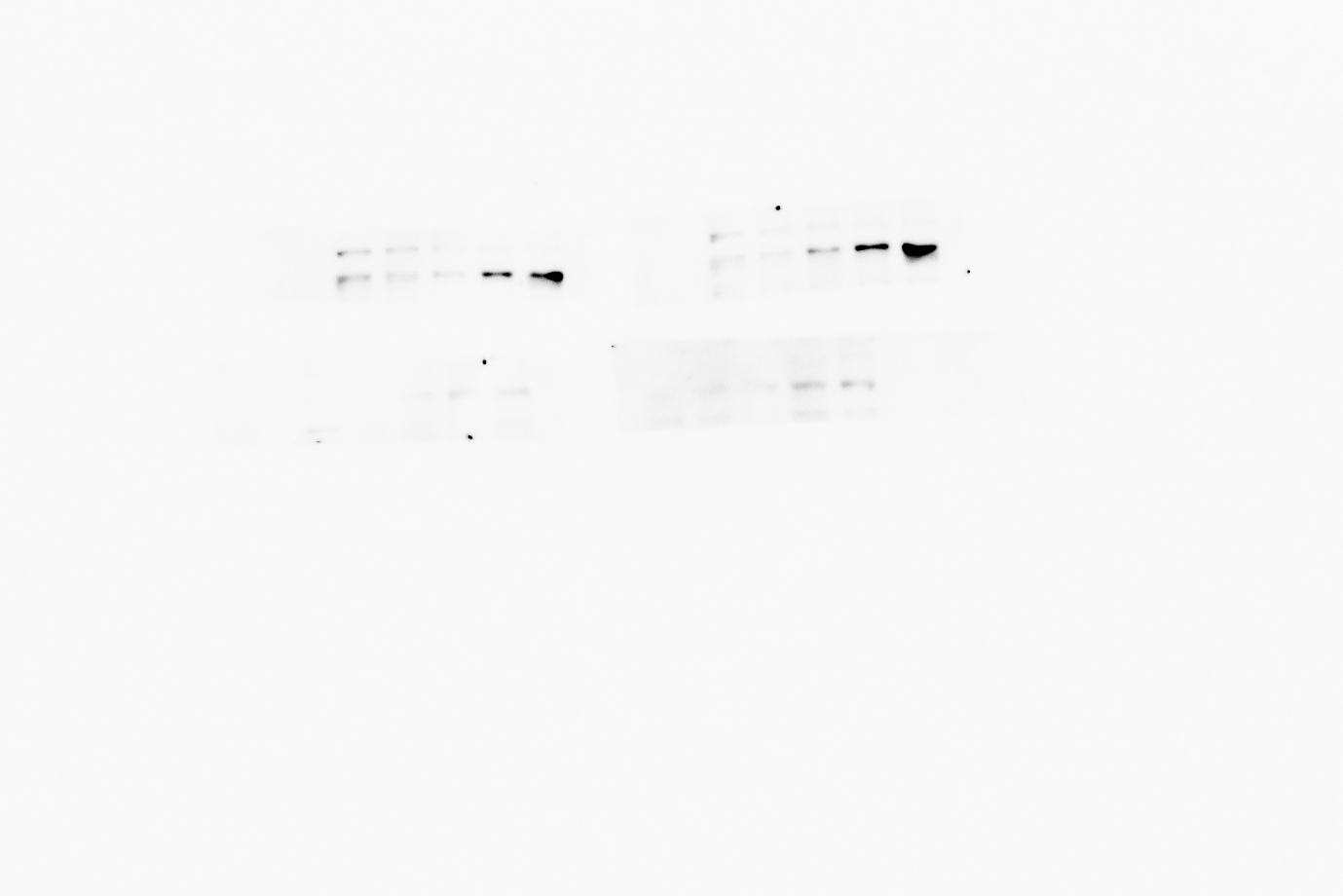


LaminB (Nuclear fraction) (Fig. 1f)


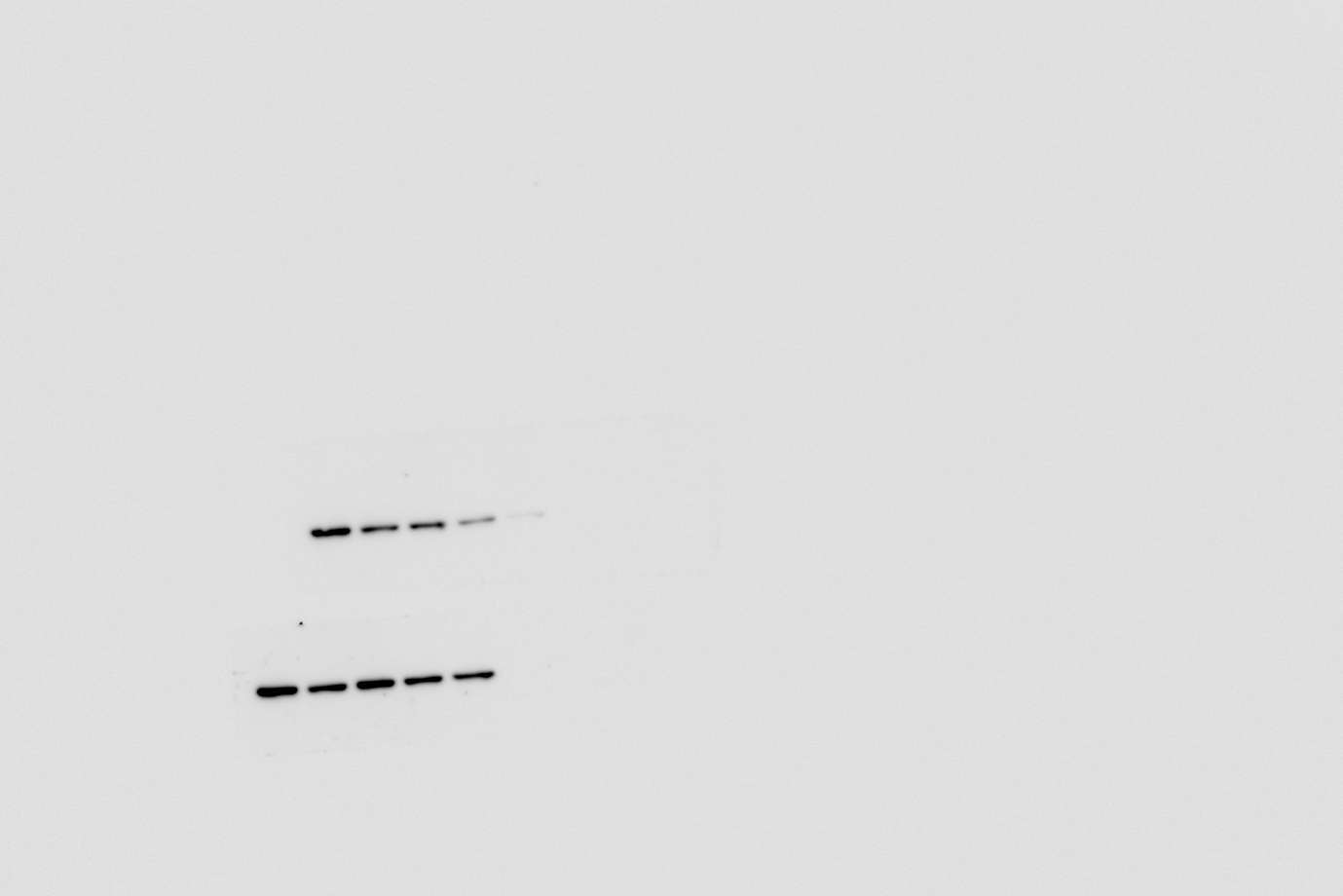


p-IκBα (Fig. 4a)


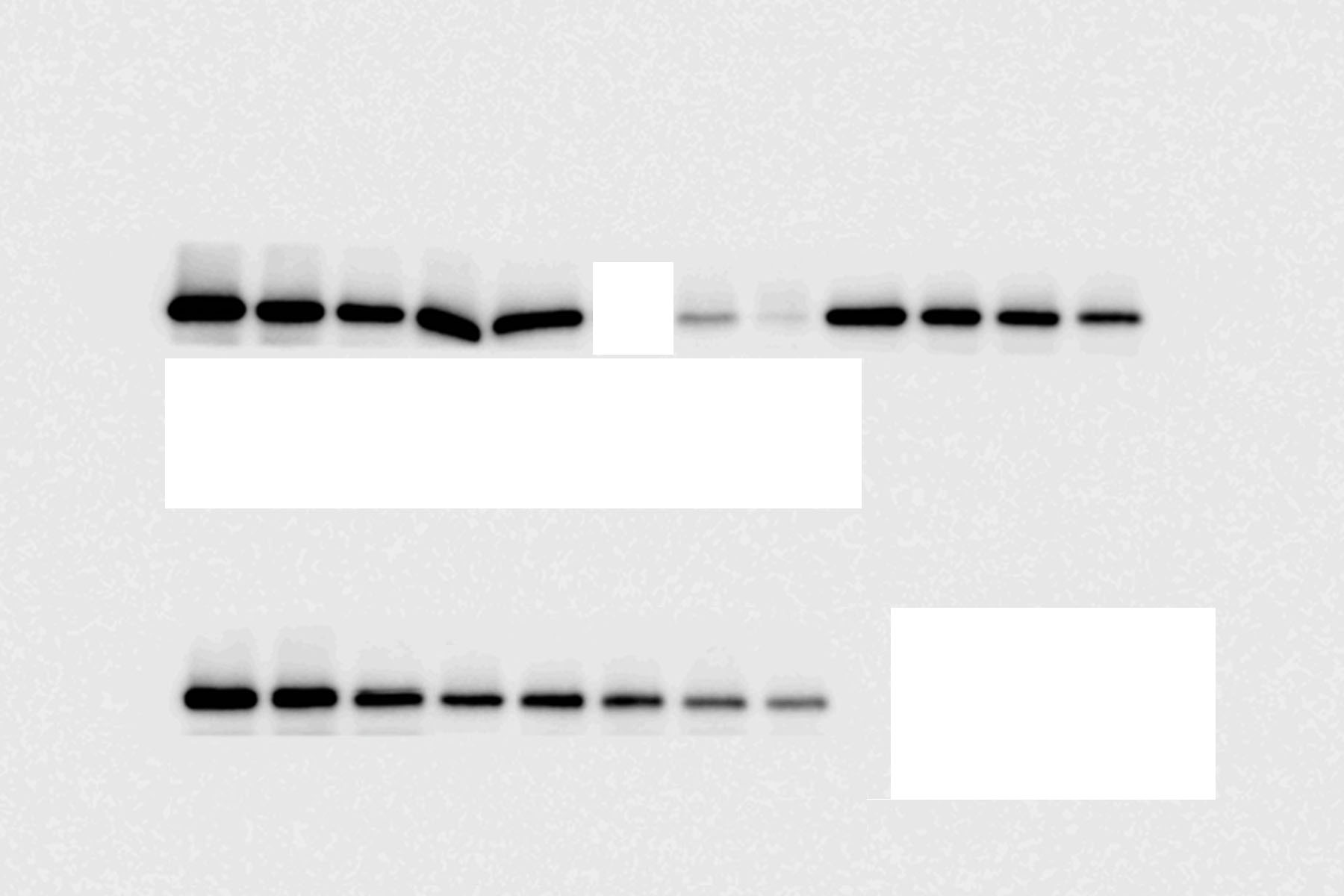


p-NFκB (Fig. 4a)


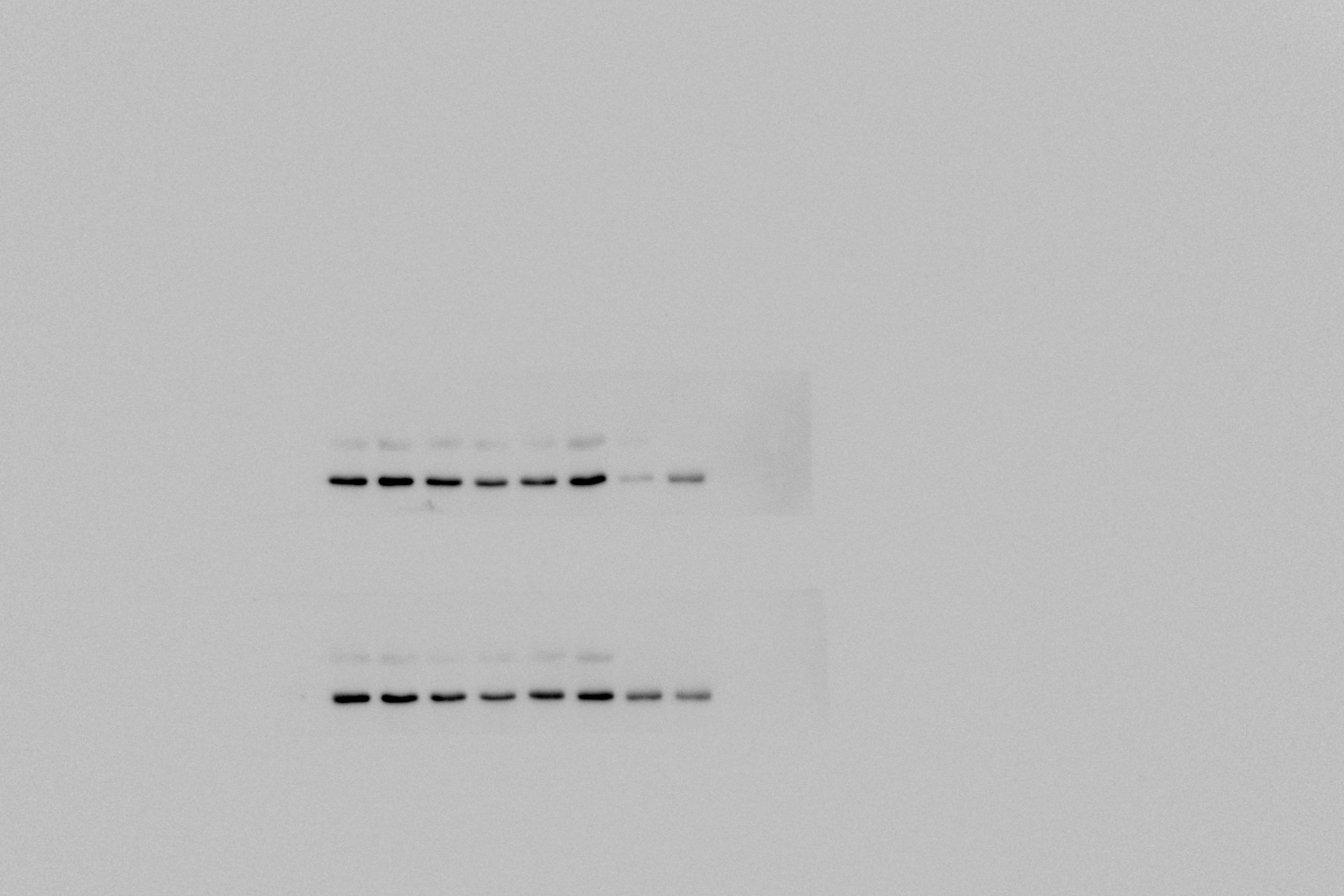


NFκB (Fig. 4a)


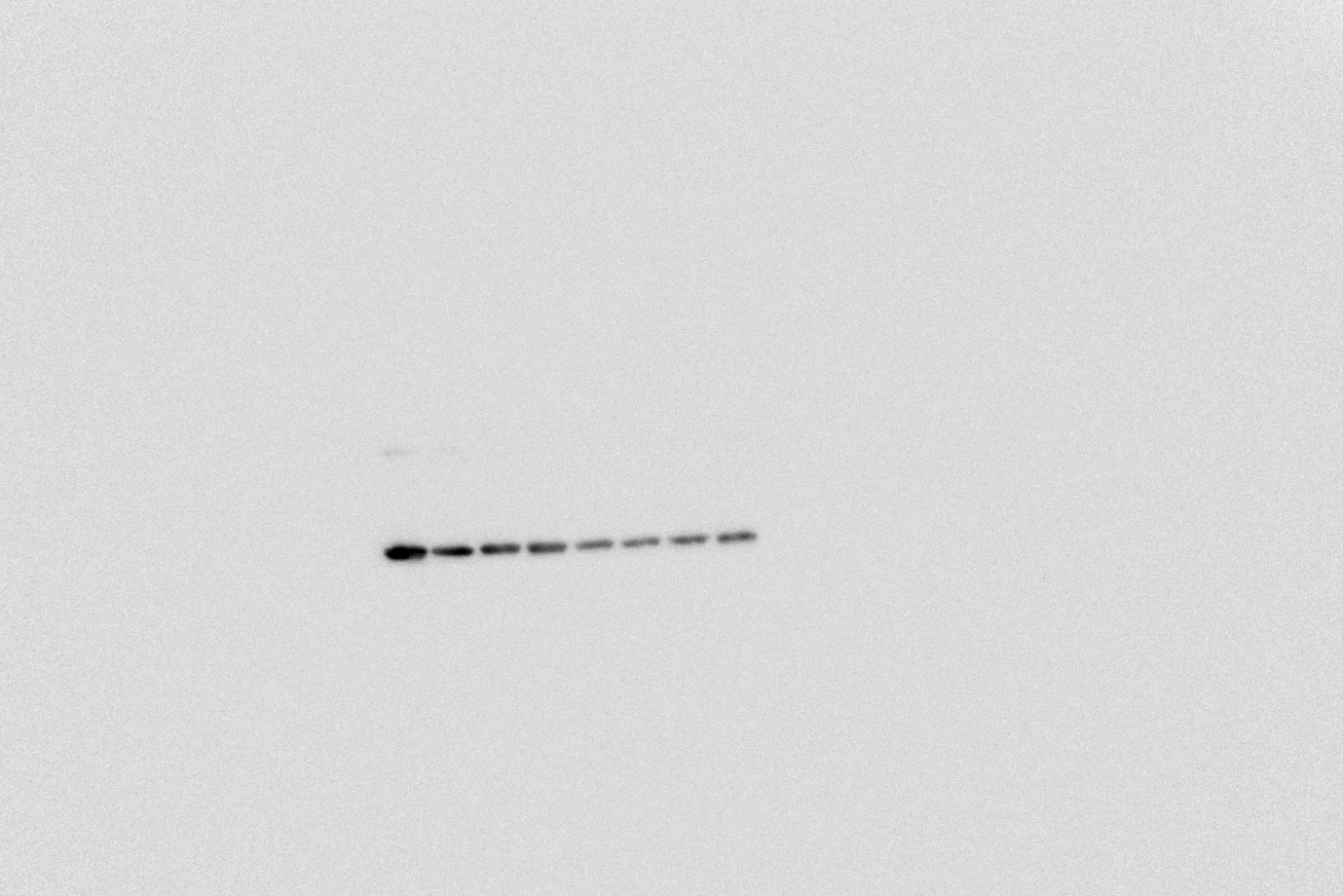


Cox2 (Fig. 4a)


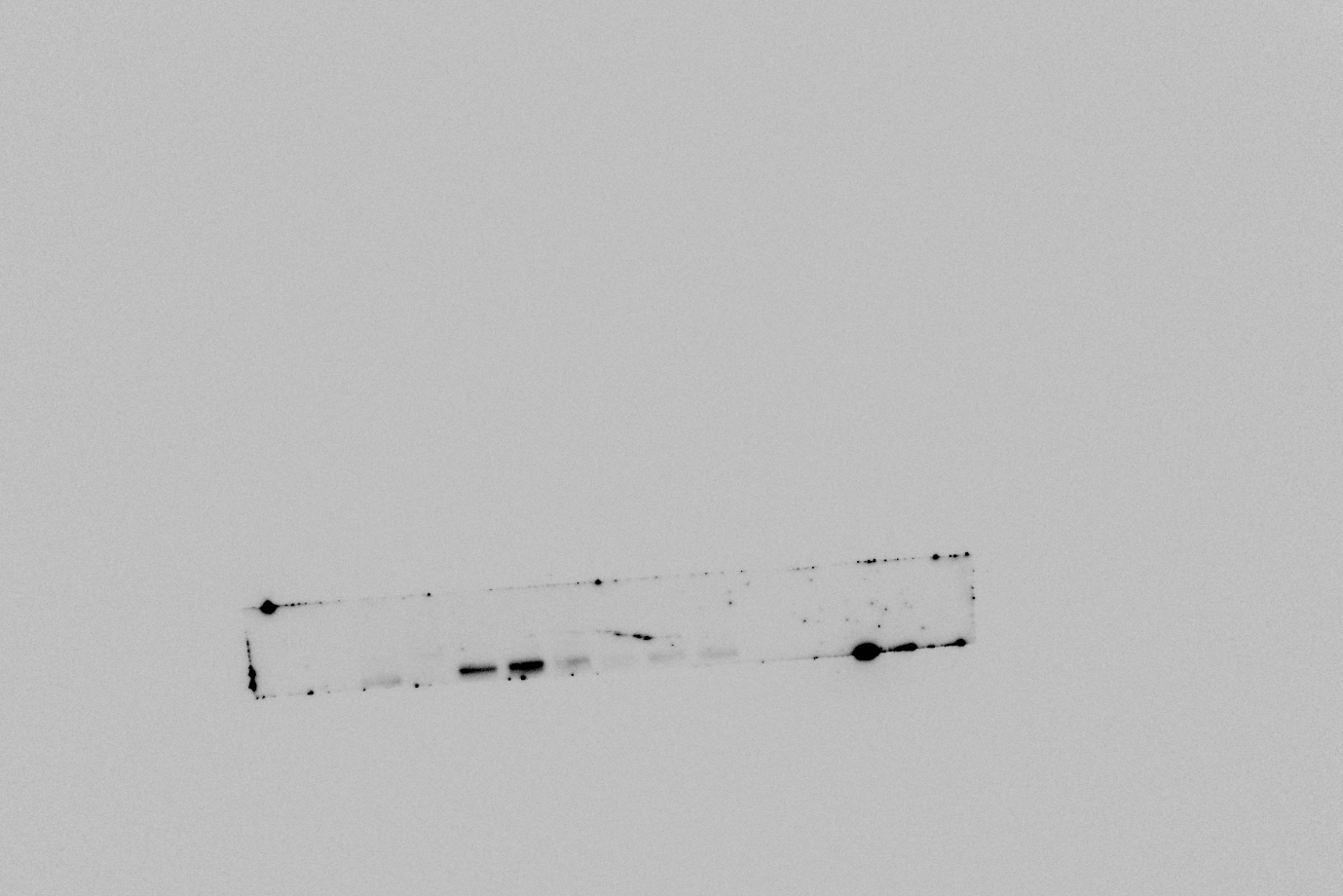


β-actin (Fig. 4a)


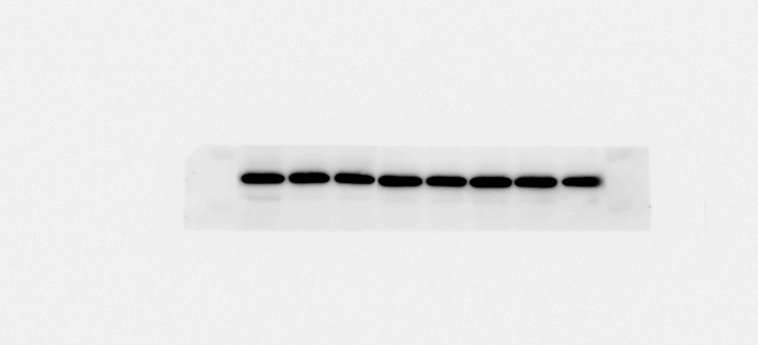


Nrf2 (Fig. 4c)


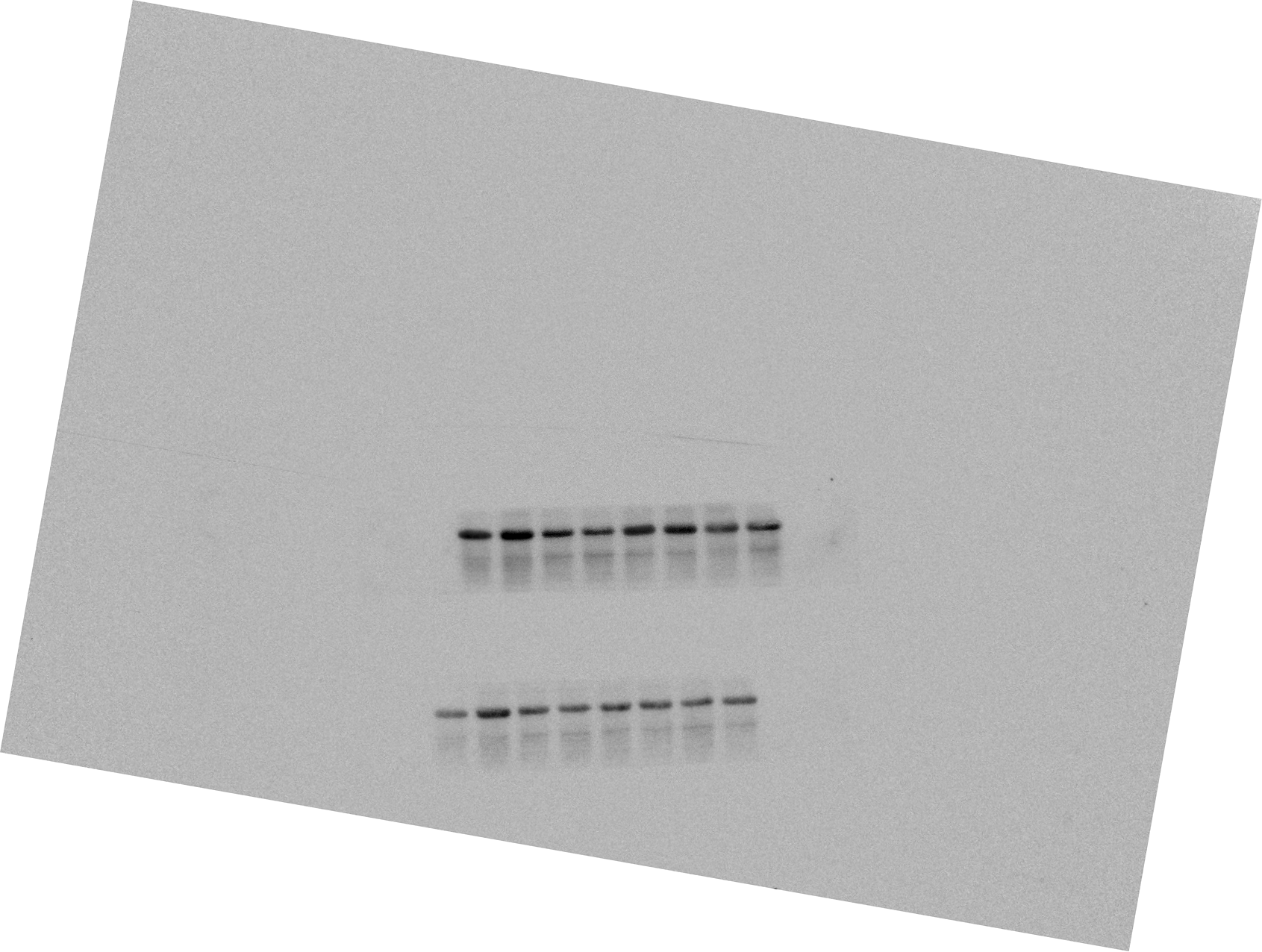


HO-1 (Fig. 4c)


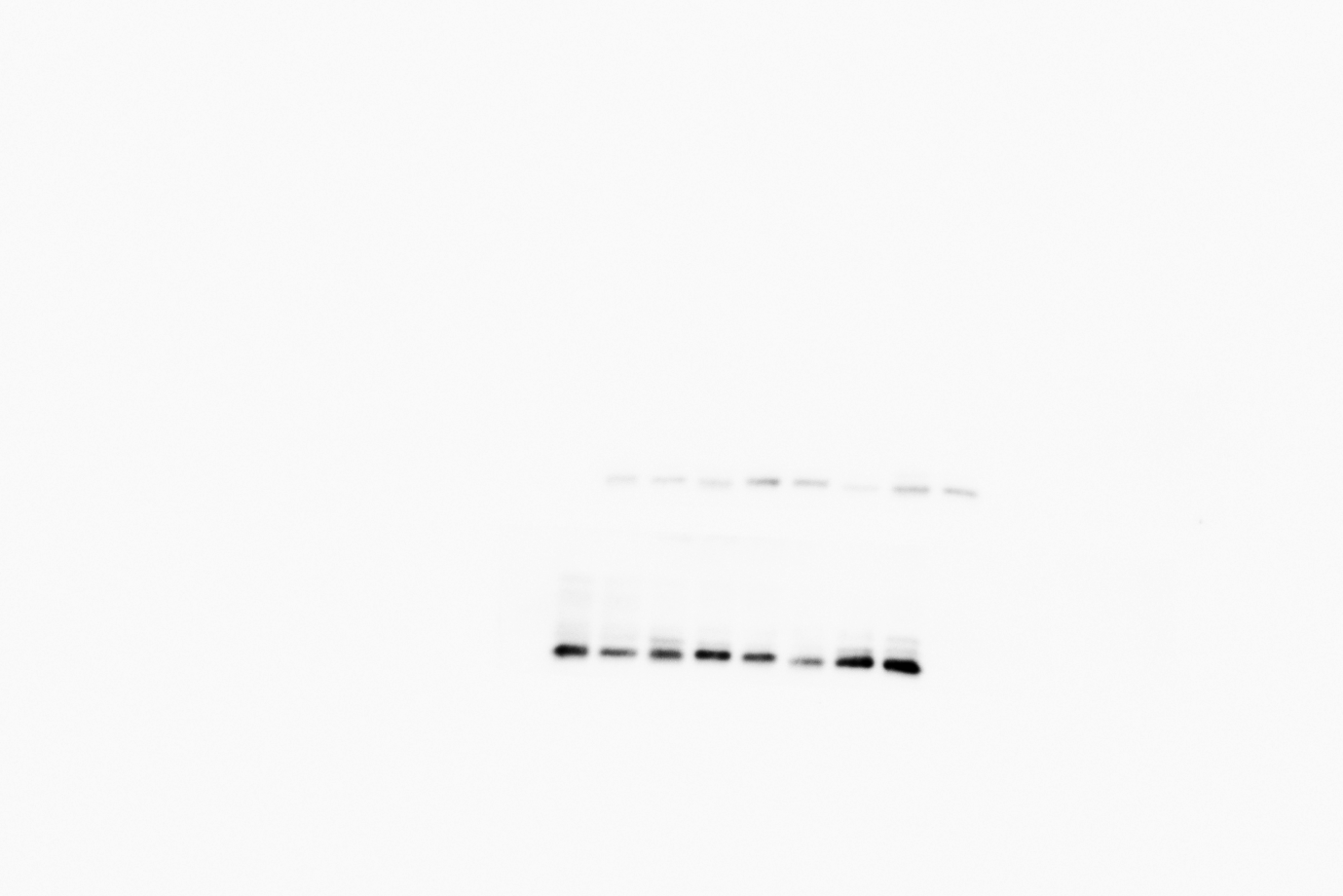


β-actin (Fig. 4c)


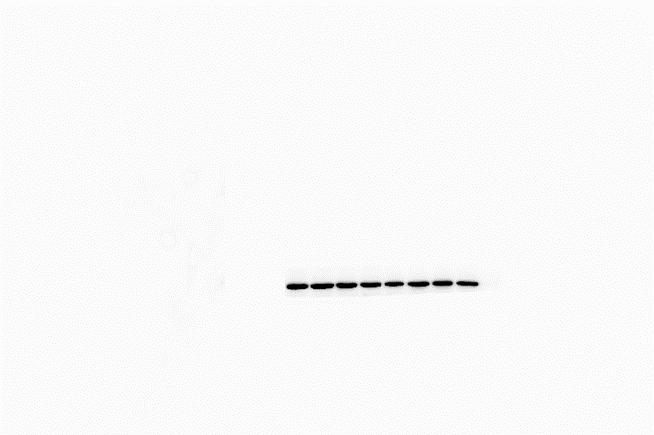


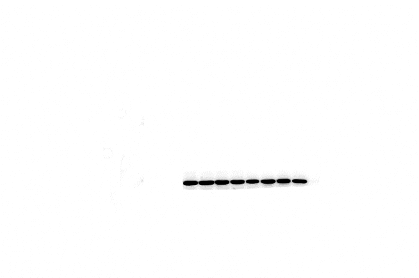


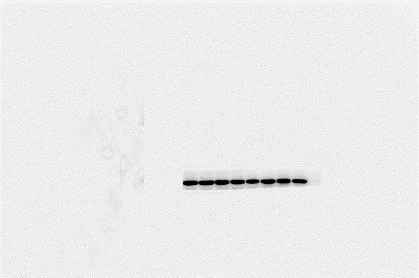


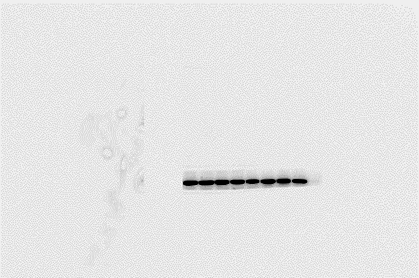

Supplement: Supplementary file 4 — Additional file 4. [file 12906_2022_3536_MOESM4_ESM.docx]
